# Dual TLR2/9 Recognition of Herpes Simplex Virus Infection Is Required for Recruitment and Activation of Monocytes and NK Cells and Restriction of Viral Dissemination to the Central Nervous System

**DOI:** 10.3389/fimmu.2018.00905

**Published:** 2018-04-30

**Authors:** Erdenebileg Uyangaa, Jin Young Choi, Ajit Mahadev Patil, Ferdaus Mohd Altaf Hossain, Sung OK Park, Bumseok Kim, Koanhoi Kim, Seong Kug Eo

**Affiliations:** ^1^College of Veterinary Medicine and Bio-Safety Research Institute, Chonbuk National University, Iksan, South Korea; ^2^Faculty of Veterinary, Animal and Biomedical Sciences, Sylhet Agricultural University, Sylhet, Bangladesh; ^3^Department of Pharmacology, School of Medicine, Pusan National University, Yangsan, South Korea

**Keywords:** toll-like receptor, herpes simplex virus, monocytes, NK cells, dendritic cells

## Abstract

The importance of TLR2 and TLR9 in the recognition of infection with herpes simplex virus (HSV) and HSV-caused diseases has been described, but some discrepancies remain concerning the benefits of these responses. Moreover, the impact of TLR2/9 on innate and adaptive immune responses within relevant mucosal tissues has not been elucidated using natural mucosal infection model of HSV. Here, we demonstrate that dual TLR2/9 recognition is essential to provide resistance against mucosal infection with HSV *via* an intravaginal route. Dual TLR2/9 ablation resulted in the highly enhanced mortality with exacerbated symptoms of encephalitis compared with TLR2 or TLR9 deficiency alone, coinciding with highly increased viral load in central nervous system tissues. TLR2 appeared to play a minor role in providing resistance against mucosal infection with HSV, since TLR2-ablated mice showed higher survival rate compared with TLR9-ablated mice. Also, the high mortality in dual TLR2/9-ablated mice was closely associated with the reduction in early monocyte and NK cell infiltration in the vaginal tract (VT), which was likely to correlate with low expression of cytokines and CCR2 ligands (CCL2 and CCL7). More interestingly, our data revealed that dual TLR2/9 recognition of HSV infection plays an important role in the functional maturation of TNF-α and iNOS-producing dendritic cells (Tip-DCs) from monocytes as well as NK cell activation in VT. TLR2/9-dependent maturation of Tip-DCs from monocytes appeared to specifically present cognate Ag, which effectively provided functional effector CD4^+^ and CD8^+^ T cells specific for HSV Ag in VT and its draining lymph nodes. TLR2/9 expressed in monocytes was likely to directly facilitate Tip-DC-like features after HSV infection. Also, dual TLR2/9 recognition of HSV infection directly activated NK cells without the aid of dendritic cells through activation of p38 MAPK pathway. Taken together, these results indicate that dual TLR2/9 recognition plays a critical role in providing resistance against mucosal infection with HSV, which may involve a direct regulation of Tip-DCs and NK cells in VT. Therefore, our data provide a more detailed understanding of TLR2/9 role in conferring antiviral immunity within relevant mucosal tissues after mucosal infection with HSV.

## Introduction

Herpes simplex viruses including type 1 (HSV-1) and type 2 (HSV-2) are ubiquitous, host-adapted pathogens with a prevalence rate of around 90% worldwide (1, 2). Notably, infection with HSV-1 and HSV-2 *via* a genital route is the most frequent cause of genital ulceration and results in a lifelong latent infection of the host after peripheral replication in genital tissues (1, 2). This latent infection evokes a relapsing pattern of illness in patients, which has an impact on physiological and social quality of life, as well as increasing the opportunity for other infections such as human immunodeficiency virus (3–5). However, currently there is no available vaccine to prevent initial infection, although pharmacological interventions, such as acyclovir, are used for the treatment of HSV-related symptoms (6).

TLRs expressed on the cell surface and within endosomes of dendritic cells (DCs), NK cells, and other innate immunity-related cellular components are key sensors of viral infection leading to activation of innate and adaptive immune responses. HSV glycoproteins including gH/gL and gB are likely to bind and activate TLR2 on the cell membrane that induces NF-κB activation and cytokine production for initiating innate immune responses (7, 8). Endosomal TLR9 also plays an important role in detecting HSV DNA, thereby leading to TLR9-dependent production of cytokines and type I IFN (IFN-I) in specialized plasmacytoid DCs (pDCs) (9, 10). Furthermore, replication and transcription of HSV DNA lead to the accumulation of intermediate dsRNA that are sensed by TLR3 (11). However, the roles of these TLRs in the progression of diseases caused by HSV infection have been shown with various and different results, depending on disease models, virus strains, and inoculation routes. TLR2 provides a detrimental effect on HSV-caused encephalitis through inducing CCL2 production in the brain after intraperitoneal inoculation with HSV-1 (12). TLR2 has also been reported to promote the production of cytokines and chemokines in primary microglia after HSV-1 infection (13). These results suggest that TLR2 plays a role in the immunopathology of HSV infection. Similarly, the ablation of TLR2 and, to a lesser extent, TLR9 results in significantly diminished lesions in stromal keratitis caused by HSV-1 infection (14). By contrast with these findings, TLR2 appears to play a role in reducing viral load in the trigeminal ganglia or brain after intravaginal (i.vag.) infection with HSV-2, and such control of viral replication requires TLR9 for maximal synergy (15). Also, the ablation of TLR9 and TLR2/9 results in highly increased susceptibility to HSV-caused encephalitis after intranasal inoculation with HSV-1 (16), which suggests that TLR2/9 are required for preventing HSV dissemination into central nervous system (CNS) tissues. These various results on the role of TLR2/9 in HSV-caused diseases indicate the need for detailed analysis in a more relevant infection model for a clear understanding. Furthermore, the impact of TLR2/9 on early innate immune responses and subsequent adaptive immunity within the relevant mucosal sites after mucosal infection with HSV has not been addressed. Knowledge of the role of TLR2/9 in generating innate immunity within the relevant mucosal sites is needed for prophylactic and therapeutic approaches.

A murine model of HSV-1 or HSV-2 infection *via* the i.vag. route has been frequently used as a naturally relevant model for genital infection since most natural infections with HSV begin by invasion in local peripheral mucosal tissues such as the vaginal tract (VT). Mice appear to be susceptible to i.vag. infection with HSV-1 or HSV-2 only during the catabolic metestrous-2 and diestrous stages of the estrous cycle (17–20). Thus, treatment of mice with progesterone (DepoProvera^®^) is required for consistent i.vag. infection with HSV in order to maintain the mice at the diesterous-like stage. Upon entering the mucosal surface of the VT, HSV replicates initially within the epithelial layer of the VT and then spreads into the CNS upon retrograde transport of virions into the sacral ganglia, resulting in fatal paralysis (21, 22). Studies using a murine model of genital herpes have shown that robust IFN-γ-producing T-helper 1 (Th1) CD4^+^ and CD8^+^ T-cell immunity is essentially required for protection against primary and secondary HSV-1 or HSV-2 infection *via* the VT (23, 24). Also, the importance of innate immune responses, including NK cells, monocytes, and neutrophils, as well as the production of IFN-I, IL-12, and IL-18 in suppressing viral replication and reducing virus-mediated mortality has been demonstrated (21). Among innate immune cells, NK cells appear to play a critical role in HSV control by recognizing and killing infected cells upon engagement of NK cell-activating receptors with the putative ligands expressed on the infected cells or the loss of inhibitory signals due to missing self (25, 26). The coordinate role of CD11b^+^Ly-6C^hi^ monocytes in conferring protection against mucosal infection with HSV has also been described (27). Ly-6C^hi^ monocytes emigrate from bone marrow (BM) into the blood stream in a CCR2-dependent manner upon pathogenic infection, and then are recruited in inflamed tissues (28). Ly-6C^hi^ monocytes have been shown to give rise to Ly-6C^hi^ monocyte-derived cells (MCs) including monocyte-derived DCs, monocyte-derived macrophage, or myeloid-derived suppressor cells (29). Notably, TNF-α and iNOS-producing dendritic cells (Tip-DCs) that produce TNF-α and iNOS are a subset of monocyte-derived DCs. It was suggested that Tip-DCs could be called iNOS^+^ MCs to underline both their monocytic origin and their iNOS-mediated killing capabilities (29). Tip-DCs have been shown to play an important role in lysis and clearance of various pathogens, including *Listeria monocytogens* (30) and *Toxoplasma gondii* (31). Likewise, in genital infection model of HSV, Ly-6C^hi^ monocytes are CCR2-dependently recruited into inflamed tissues, where they exert tailored protective immunity through stimulating antiviral Th1 CD4^+^ T-cell immunity (32). NK cells and Ly-6C^hi^ monocytes have been shown to express multiple TLRs (33, 34). NK cells have been found to be activated by TLR ligands derived from pathogens in DC–NK crosstalk or direct stimulation manner (33). Also, the maturation of functional Tip-DCs from Ly-6C^hi^ monocytes is likely to depend on the adaptor molecule MyD88 (35). These results suggest that TLR signaling plays an important role in functional maturation of Tip-DCs and NK cells. Nevertheless, the impact of TLR2/9 on functional maturation of Tip-DCs and NK cells and subsequent Ag-specific T-cell responses within relevant mucosal tissues after mucosal infection with HSV-1 or HSV-2 has not yet been addressed.

Here, we discovered that dual TLR2/9 recognition is essential for functional maturation of Ly-6C^hi^ monocyte-derived Tip-DCs and NK cells in primary inflamed tissue, such as the VT, after mucosal infection with HSV-1. TLR2/9-dependent maturation of Tip-DCs from Ly-6C^hi^ monocytes was required to specifically present cognate Ag for CD4^+^ and CD8^+^ T-cell responses in the VT and its draining LNs. Moreover, our data revealed that dual TLR2/9 recognition directly activated NK cells through activation of the p38 MAPK pathway. This TLR2/9-dependent activation of Tip-DCs and NK cells was closely associated with the protective immunity against mucosal infection with HSV-1. Collectively, our data provide a more detailed understanding for the role of TLR2/9 in conferring protective immunity in mucosal tissues after mucosal infection with HSV.

## Materials and Methods

### Ethics Statement

All animal experiments described in this study were conducted at Chonbuk National University according to the guidelines set by the Institutional Animal Care and Use Committee (IACUC) of Chonbuk National University, and were pre-approved by the Ethical Committee for Animal Experiments of Chonbuk National University (Permission code 2013-0028). The animal research protocol used in this study followed the guidelines set up by the nationally recognized Korea Association for Laboratory Animal Sciences (KALAS). All experimental protocols requiring biosafety were approved by the Institutional Biosafety Committee (IBC) of Chonbuk National University.

### Animals, Cells, Viruses, and *In Vivo* Viral Infection

Female C57BL/6 (H-2^b^) mice (5–6 weeks old) were purchased from Samtako Co. (O-San, Korea), and TLR2 or TLR9-deficient (TLR2 KO or TLR9 KO, respectively) mice (H-2^b^) have been described elsewhere (36). OT-II mice, which are transgenic for the V_α2_/V_β5_ TCR that recognizes the I-A^b^-restricted peptide (OVA_323–339_, ISQAVHAAHAEINEAGR) of chicken ovalbumin (OVA), were obtained from The Jackson Laboratory (Bar Harbor, ME, USA). TLR2/9 double knock-out (TLR2/9 DKO) mice were generated by crossing TLR2 KO and TLR9 KO mice at Chonbuk National University. All mice were genotyped and bred in the animal facilities of Chonbuk National University. HSV-1 McKrae and KOS strains were propagated in Vero cells (CCL81; ATCC, Manassas, VA, USA) using DMEM supplemented with 2.5% FBS, penicillin (100 U/ml), and streptomycin (100 U/ml). The virus stocks were stored in aliquots at −80°C after titration with a conventional plaque assay using Vero cells. For an *in vivo* viral challenge, mice were previously treated with progesterone to synchronize their estrous cycles, as described earlier (17–20). Briefly, mice were subcutaneously injected with Depo-Provera (DP) (Sigma-Aldrich) at 2 mg per mouse. Five days following the injection of DP, the mice were challenged i.vag. with different doses of the HSV-1 McKrae strain (1 × 10^6^, 1 × 10^7^, and 5 × 10^7^ PFU/mouse). Infected mice were examined daily for vaginal inflammation, neurological illness, and death, as described previously (37). Mice were scored from 1 to 5 depending on the clinical severity of disease: 0, no change; 1, mild inflammation; 2, moderate swelling; 3, severe inflammation; 4, paralysis; and 5, death.

### Antibodies and Reagents

The mAbs used for flow cytometric analysis and other experiments were obtained from eBioscience (San Diego, CA, USA) or BD Biosciences (San Diego, CA, USA) (Table S1 in Supplementary Material). The peptides of the defined H-2K^b^-restricted epitope HSV-1 gB_498-505_ (SSIEFARL) and I-A^b^-restricted epitope OVA_323-339_ (ISQAVHAAHAEINEAGR) peptides were chemically synthesized by Peptron Inc. (Daejeon, Korea). Lipopolysaccharide (LPS) was purchased from Sigma-Aldrich (St. Louis, MO, USA). Primers specific for HSV-1 gB, cytokines, and chemokines (Table S2 in Supplementary Material) were synthesized by Bioneer Corp. (Daejeon, Korea), and were used for PCR amplification of target genes.

### Quantitation of Viral Burden and Cytokine/Chemokine Expression

#### Quantitative Real-Time PCR for Viral Burden and Cytokine/Chemokine Expression

Viral burden was determined by real-time qPCR using genomic DNA extracted from collected tissues, whereas the expression of cytokines (TNF-α, IL-6, IL-23, and IFN-β), iNOS, and chemokines (CCL2, CCL3, CXCL1, and CXCL2) was assessed by real-time qRT-PCR using total RNA extracted from collected tissues and sorted cells. Mice were infected i.vag. with HSV-1 McKrae (1 × 10^7^ PFU/mouse) and tissues including the VT, iliac LNs (ILNs), spleen (Spl), brain, and spinal cord (SC) were harvested at 0, 2, and 5 days post-infection (dpi). Following the extraction of genomic DNA or total RNA, real-time qPCR using a CFX96™ Real-Time PCR Detection System (Bio-Rad Laboratories, Hercules, CA, USA) was employed. To determine the expression of cytokine and chemokine mRNAs, reverse transcription of total RNAs was performed with High-Capacity cDNA Reverse Transcription Kits (Applied Biosystems, Foster City, CA, USA) before real-time qPCR. The reaction mixture contained 2 µl of template cDNA, 10 µl of 2 × SYBR Green based real-time RT-PCR, and 200 nM of specific primers at a final volume of 20 µl. Double-stranded nucleic acids were denatured at 95°C for 30 s and then subjected to 45 cycles of 95°C for 5 s and 60°C for 20 s. After the reaction cycle was completed, the temperature was increased from 65°C to 95°C at a rate of 0.2°C/15 s, and fluorescence was measured every 5 s to construct a melting curve. A control sample that did not contain template DNA was run with each assay, and all determinations were performed at least in duplicate to ensure reproducibility. The authenticity of the amplified product was determined by melting curve analysis. Viral burden was expressed as viral DNA copy number per microgram of genomic DNA, and the expression of cytokines and chemokines was expressed as relative fold expression compared with uninfected control group, after normalization to the housekeeping gene β-actin. All data were analyzed using the Bio-Rad CFX Manager, version 2.1 analysis software (Bio-Rad Laboratories).

#### Cytometric Bead Array (CBA)

The levels of cytokines and chemokines in vaginal lavages were measured by a cytometric bead array (CBA) specific for IL-6, TNF-α, GM-CSF, CCL2 (MCP-1), CCL3 (MIP-1α), CCL4 (MIP-1β), CCL5 (RANTES), and CCL7 (MCP-3) according to the manufacturer’s protocols (eBioscience).

### Analysis of Infiltrated Leukocytes Into the VT, ILNs, and SC

Vaginal tracts, ILNs, and SCs were collected from wild-type (WT), TLR2 KO, TLR9 KO, and TLR2/9 DKO mice infected with the HSV-1 McKrae strain (1 × 10^7^ PFU/mouse) at 0, 2, and 5 dpi. Leukocytes infiltrated in the SC and VT tissues were harvested by gently pressing them through a sterile cell strainer with 100 µm pore following digestion with 25 µg/ml of collagenase type IV (Worthington Biochem, Freefold, NJ, USA) in RPMI 1640 medium for 1 h at 37°C. Leukocytes of SCs were further separated using Optiprep density gradient (18/10/5%) centrifugation at 400 × *g* for 30 min (Axis-Shield, Oslo, Norway), after which cells were collected from 18 to 10% interface and washed twice with PBS. The ILN cells were prepared by gently pressing them through sterile cell strainers with 100 µm pore. All cells were then counted and stained for CD11b, CD11c, Ly6C, and Ly6G with directly conjugated antibodies (eBioscience) for 30 min at 4°C. In some experiments, intracellular TNF-α or iNOS staining combined with surface CD11c staining was used to detect vaginal TNF-α and iNOS-producing Tip-DCs. Finally, the cells were fixed with 10% formaldehyde. Data collection and analysis were performed with a FACSCalibur flow cytometer (Becton Dickson Medical Systems, Sharon, MA, USA) and FlowJo (Tree Star, San Carlos, CA, USA) software.

### Analysis of NK Cell Activity

The activity of NK cells was assessed by the capacity to produce IFN-γ and granzyme B (GrB) following brief stimulation with PMA and ionomycin (Sigma-Aldrich). Cells were prepared from the VTs, ILNs, and Spls of WT, TLR2 KO, TLR9 KO, and TLR2/9 DKO mice at 2 dpi, and stimulated with PMA and ionomycin (IFN-γ, PMA 50 ng/ml plus ionomycin 750 ng/ml for 2 h; GrB, PMA 50 ng/ml plus ionomycin 750 ng/ml for 4 h) in the presence of monensin (2 µM). The stimulated cells were washed twice with PBS containing monensin and surface-stained with CD3, NK1.1, and DX5 antibodies for 30 min at 4°C. After fixation, the cells were washed twice with PBS, and permeabilized with 1× Permeabilization buffer (eBioscience). The cells were then subjected to intracellular IFN-γ and GrB staining in permeabilization buffer for 30 min at room temperature. Then, the stained cells were washed twice with permeabilization buffer and FACS buffer, and analysis was performed with a FACSCalibur flow cytometer (Becton Dickson Medical Systems) using FlowJo (Tree Star) software.

### Histopathological Examinations and Confocal Microscopy

For histopathological examinations, vaginal tissues derived from WT, TLR2 KO, TLR9 KO, and TLR2/9 DKO mice were embedded in paraffin 0 and 2 days following HSV-1 infection (1 × 10^7^ PFU/mouse), and 10-µm sections were prepared and stained with H&E. Sections were analyzed using a Nikon Eclipse E600 microscope (Nikon, Tokyo, Japan). For confocal microscopy staining, VTs were collected and frozen in optimum cutting temperature compound at 0 and 2 dpi. 6–7 µm thick sections were cut, air-dried, and fixed with 1:1 mixture of acetone and methanol for 15 min at −20°C. After washing with PBS three times, non-specific binding was blocked with 10% normal goat serum and the sections were permeabilized with 0.1% Triton X-100. Staining was performed by incubating the sections overnight in a moist chamber at 4°C with PE-Ly-6C, biotin-conjugated anti-mouse myeloid-derived cell marker CD11b, DC marker CD11c, and NK cell marker DX5 plus anti-HSV-1 gB (Abcam). Primary antibodies were detected with secondary FITC-conjugated goat anti-rabbit IgG and PE-conjugated streptavidin. Nuclei were counterstained with DAPI (4′,6-diamidino-2-phenylindole) (Sigma-Aldrich). Finally, the fluorescence was observed by a confocal laser scanning microscope (Cal Zeiss, Zena, Germany).

### Ag-Presentation Capability of Sorted Ly-6C^hi^ Monocytes

The Ag-presentation capability of Ly-6C^hi^ monocytes was assessed by measuring IL-2 production and viable ATP bioluminescence in response to stimulation of OT-II CD4^+^ T cells with an epitope peptide. Briefly, OVA_323-339_-specific CD4^+^ T cells were purified from OT-II mice using a MACS LS column (Miltenyi Biotec) according to the manufacturer’s instructions. The purified OT-II CD4^+^ T cells were then co-cultured with vaginal Ly-6C^hi^ monocytes sorted from WT, TLR2 KO, TLR9 KO, and TLR2/9 DKO mice in the presence of the OVA_323-339_ epitope peptide for 72 h at 37°C. The proliferated cells were then evaluated using a Vialight cell proliferation assay kit (Cambrex Bio Science) according to the manufacturer’s instructions. The level of IL-2 in culture media was determined by a cytokine ELISA.

### HSV Ag-Specific CD4^+^and CD8^+^ T-Cell Responses

Herpes simplex virus Ag-specific CD4^+^ and CD8^+^ T-cell responses were determined by intracellular CD154 staining (38), combined with intracellular IFN-γ or TNF-α staining in response to stimulation with UV-inactivated HSV or HSV gB_498-505_ epitope, respectively. Briefly, cells were prepared from the VTs and ILNs of surviving WT, TLR2 KO, TLR9 KO, and TLR2/9 DKO mice 7 dpi. Erythrocytes were depleted by treating single-cell suspensions with ammonium chloride-containing Tris buffer (NH_4_Cl-Tris) at 37°C for 20 min. Then, cells were co-cultured with UV-inactivated HSV-1 KOS-pulsed APCs (5:1 ratio) in the presence of PE-conjugated CD154 antibody for 12 h at 37°C. HSV-1 Ag-specific CD4^+^ T cells were enumerated by intracellular IFN-γ and TNF-α staining combined with surface CD4 staining. CD8^+^ T-cell responses specific for the HSV gB_498-505_ epitope were determined by 8 h-stimulation with the gB_498-505_ epitope peptide followed by intracellular IFN-γ and TNF-α staining combined with surface CD8 staining. Monensin (2 µM) was added to the antigen-stimulated cells 6 h before harvesting. Finally, the stained cells were analyzed with a FACSCalibur flow cytometer (Becton Dickson Medical Systems) using FlowJo (Tree Star) software.

### *In Vitro* Tip-DC-Like Maturation of Ly-6C^hi^ Monocytes and NK Cell Activation

CD11b^+^Ly-6C^hi^ monocytes and CD3^-^NK1.1^+^DX5^+^ NK cells were purified from the Spls of WT, TLR2 KO, TLR9 KO, and TLR2/9 DKO mice *via* flow cytometry sorting on a FACSAria. Then, fresh CD11b^+^Ly-6C^hi^ monocytes and NK cells were stimulated with HSV-1 (5.0 moi) for 24 h at 37°C. The phenotypic maturation or activation of Tip-DCs from Ly-6C^hi^ monocytes and NK cells was evaluated by cell surface staining with specific Abs for phenotypic markers or intracellular IFN-γ staining, respectively. In some experiments, total RNA extracted from stimulated Ly-6C^hi^ monocytes was employed in real-time qRT-PCR.

### Co-Culture Experiment for NK Cell–DC Crosstalk

Dendritic cells were generated from BM cells as described previously (39, 40). Briefly, BM cells harvested from femurs and tibiae of WT, TLR2 KO, TLR9 KO, and TLR2/9 DKO mice were cultured in RPMI 1640 supplemented with mouse GM-CSF (2 ng/ml) and IL-4 (10 ng/ml). The cultures were replenished with fresh media containing growth factors on day 4. On day 7, CD11c^+^ DCs were harvested for NK cell stimulation. Co-culture of NK cells–DC was performed as described with some modifications (41). Briefly, CD3^-^NK1.1^+^DX5^+^NK cells were purified from the Spls of WT, TLR2 KO, TLR9 KO, and TLR2/9 DKO mice *via* flow cytometry sorting on a FACSAria. Then, NK cells (1.5 × 10^5^) were co-cultured with CD11c^+^ DC (7.5 × 10^4^) at an NK cell:DC ratio of 2:1. The co-culture was subsequently stimulated with HSV-1 (5.0 moi) or LPS (200 ng/ml) for 48 h at 37°C.

### Western Blot Analysis

CD3^-^NK1.1^+^DX5^+^ NK cells were purified from the Spls of WT, TLR2 KO, TLR9 KO, and TLR2/9 DKO mice, and then stimulated with live HSV-1 (5.0 moi) for 48 h at 37°C. NK cells were lysed in PRO-PREP supplemented with protease inhibitors (iNtRON, INC., Daejeon, Korea) and resolved by electrophoresis on 10, 12, and 15% SDS-polyacrylamide gels. Samples (30 µg) were resolved by electrophoresis on 10–12.5% SDS-polyacrylamide gels. After proteins were transferred to PVDF Immobilon-P Transfer Membranes (Millipore, Billerica, MA, USA), blots were blocked with 5% non-fat dried milk or 3% BSA overnight at 4°C, and probed with the following panel of primary antibodies: rabbit anti-p44/42 MAPK, phospho-p44/42 MAPK (Thr202/Tyr204), anti-p38 MAPK, phospho-p38 (Thr180/Tyr182), anti-Akt, and phospho-Akt (Ser473) (Cell Signaling, Danvers, MA, USA). Western blots were incubated with peroxidase-conjugated secondary antibodies (SouthernBiotech, Birmingham, AL, USA) and visualized with WEST-ZOL Plus Immunoblotting detection reagents (iNtRON Biotech) using a chemi-documentation system (Fusion Fx7, Vilber Lourmat, Cedex1, France). The intensities of western blot bands were quantified by luminescence intensity of each band using Bioprofil software (Bio-1D ver.15.01, Vilber Lourmat).

### Statistical Analysis

All data were expressed as the average ± SEM, and statistically significant differences between groups were analyzed by an unpaired two-tailed Student’s *t*-test for *ex vivo* experiments and immune cell analysis. For multiple comparisons, statistical significance was determined using one-way or two-way ANOVA with repeated measures, both followed by Bonferroni *post hoc* tests. The statistical significance of viral burden and *in vivo* cytokine gene expression were evaluated by the Mann–Whitney test or unpaired two-tailed Student’s *t*-test. Kaplan–Meier survival curves were analyzed by the log-rank test. A *p*-value ≤0.05 was considered significant. All data were analyzed using GraphPadPrism4 software (GraphPad Software, Inc., San Diego, CA, USA).

## Results

### Dual Ablation of TLR2/9 Highly Enhances the Susceptibility to Mucosal HSV-1 Infection *via* Promoting CNS-Invasion of Virus

**Figure 1:**
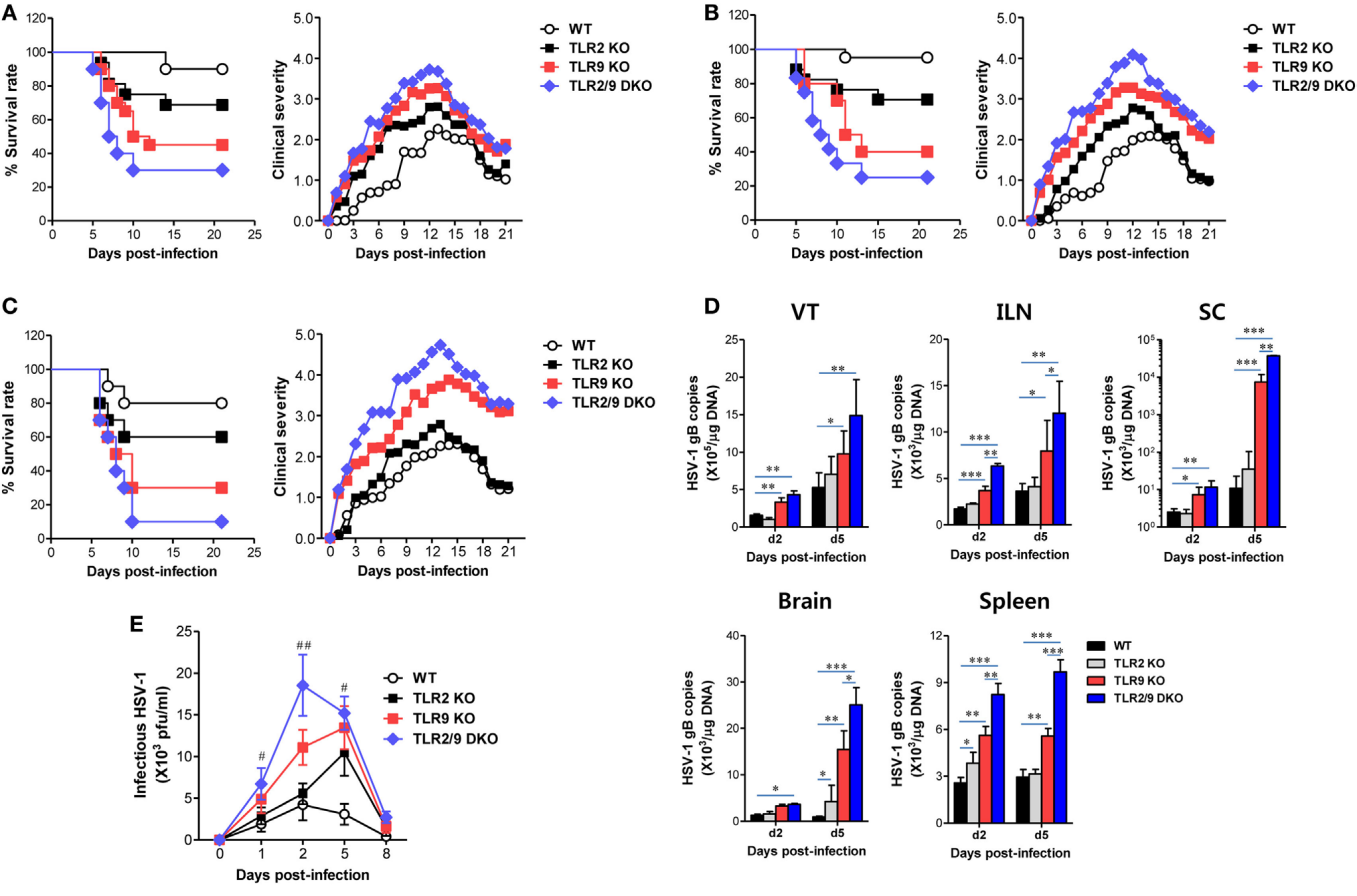
Susceptibility and viral burden of TLR2 KO, TLR9 KO, and TLR2/9 double knock-out (TLR2/9 DKO) mice against mucosal herpes simplex virus (HSV)-1 infection. **(A–C)** Survival rate and clinical score. Wild-type (WT) (C57BL/6), TLR2 KO, TLR9 KO, and TLR2/9 DKO mice (*n* = 9–12) were challenged intravaginal with different doses of the HSV-1 McKrae strain [**(A)**: 1 × 10^6^, **(B)**: 1 × 10^7^, and **(C)**: 5 × 10^7^ pfu/mouse]. Surviving mice were examined daily up to 21 days post-infection (dpi), and the clinical score was recorded at the indicated dpi. **(D)** Viral burden in inflammatory and lymphoid tissues. Viral burden in the vaginal tract (VT), iliac LNs (ILNs), spinal cord (SC), brain, and spleen of WT, TLR2 KO, TLR9 KO, and TLR2/9 DKO mice infected with HSV-1 (1 × 10^7^ pfu/mouse) was assessed by real-time qPCR at the indicated dpi. The viral burden was expressed by viral DNA copy number per microgram of genomic DNA. **(E)** Infectious virus shedding in primary target tissues. Infectious virus titer was measured by a plaque-forming assay using vaginal lavages collected at the indicated dpi. Data represent the average ± SEM of the levels derived from at least three independent experiments (*n* = 3–4). Two-way ANOVA followed by Bonferroni *post hoc* testing was conducted in **(D,E)**. *p < 0.05; **p < 0.01; ***p < 0.001 comparing levels between the indicated groups; ^#^*p* < 0.05; ^##^*p* < 0.01 comparing levels between TLR9 KO and TLR2/9 DKO mice.

To clarify the role of TLR2 and TLR9 in inflamed tissues after mucosal HSV infection, we assessed the survival of WT, TLR2 KO, TLR9 KO, and TLR2/9 DKO mice following vaginal infection with different doses of the HSV-1 McKrae strain (1 × 10^6^, 1 × 10^7^, and 5 × 10^7^ pfu/mouse). Infected WT, TLR2 KO, TLR9 KO, and TLR2/9 DKO mice showed similar clinical signs, starting with generalized piloerection and vaginal inflammation followed by hindlimb paralysis and death with CNS-invasion of the virus during 4–12 dpi. However, TLR2/9 DKO mice showed the highest mortality with 75% at a dose of 1 × 10^6^, 80% at 1 × 10^7^, and 95% at 5 × 10^7^, as compared with WT mice with 5 and 20% mortality (Figures 1A–C, *left graphs*). TLR2 KO mice showed modest mortality with 30, 30, and 40%, while TLR9 KO mice exhibited apparently enhanced mortality with 55, 60, and 70%, depending on the infection dose. These results indicate that both TLR2 and TLR9 are essential to provide the resistance to mucosal infection with HSV-1, and TLR9 appears to play a more important role in providing resistance to mucosal HSV-1 infection compared with the TLR2. In support of the increased mortality rate, TLR2/9 DKO mice exhibited the highest severity in clinical signs during disease progression after mucosal HSV-1 infection (Figures 1A–C, *right graphs*). While TLR9 KO mice were observed to have an apparently increased clinical score, TLR2 KO mice showed a modestly increased score compared with WT mice. Also, we examined viral burden in primary inflamed tissues (the VT), draining LNs (ILNs), and the Spl as well as the CNS including the SC and brain. TLR2/9 DKO mice had the highest viral burden in all examined tissues, and TLR9 KO mice also showed significantly increased levels of viral burden as compared with WT mice (Figure 1D). Of note, the CNS tissue (SC) of TLR2/9 DKO and TLR9 KO mice contained viral burdens with 1,000- to 10,000-fold increased levels 5 dpi, as compared with those of WT mice. TLR2 KO mice showed slightly but not significantly increased levels of viral burden in the examined tissues. Similarly, TLR2/9 DKO mice contained the highest levels of infectious virus in vaginal lavages (Figure 1E). TLR2 KO mice showed higher levels of infectious virus in vaginal lavages at 5 dpi, compared with WT mice. Collectively, these results indicate that both TLR2 and TLR9 are essential in providing resistance against mucosal HSV-1 infection through restricting CNS-invasion of the virus. Also, our vaginal challenge data revealed that the TLR9 molecule has a dominant role in providing resistance against mucosal HSV-1 infection compared with the TLR2 molecule.

### Both TLR2/9 Are Required for Early Ly-6C^hi^ Monocyte Accumulation in Inflamed Tissues

**Figure 2:**
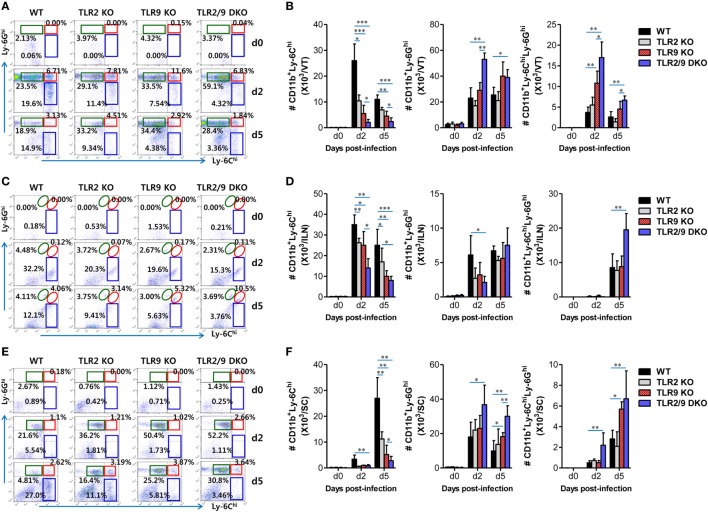
Both TLR2 and TLR9 are required for early accumulation of Ly-6C^hi^ monocytes into inflamed and lymphoid tissues following mucosal herpes simplex virus (HSV)-1 infection. Cells were prepared from the vaginal tract [VT, **(A,B)**], iliac LNs [ILNs, **(C,D)**], and spinal cord [SC, **(E,F)**] with collagenase digestion at 0, 2, and 5 days after mucosal HSV-1 infection (1 × 10^7^ pfu/mouse) and employed to determine the subcellular proportion of Ly-6C^hi^ monocytes and Ly-6G^hi^ and Ly-6C^hi^Ly-6G^hi^ neutrophils using flow cytometric analysis. The values in the dot-plots represent the average percentages of each population derived from four independent samples after gating on CD11b^+^ cells. **(A,C,E)** The frequency of each cell population. **(B,D,F)** The accumulated absolute number of each cell population. Data in the bar graphs denote the average ± SEM of the levels derived from at least three independent experiments (*n* = 3–4). Two-way ANOVA followed by Bonferroni *post hoc* testing was conducted in **(B,D,F)**. *p < 0.05; **p < 0.01; ***p < 0.001 comparing levels between the indicated groups.

**Figure 3:**
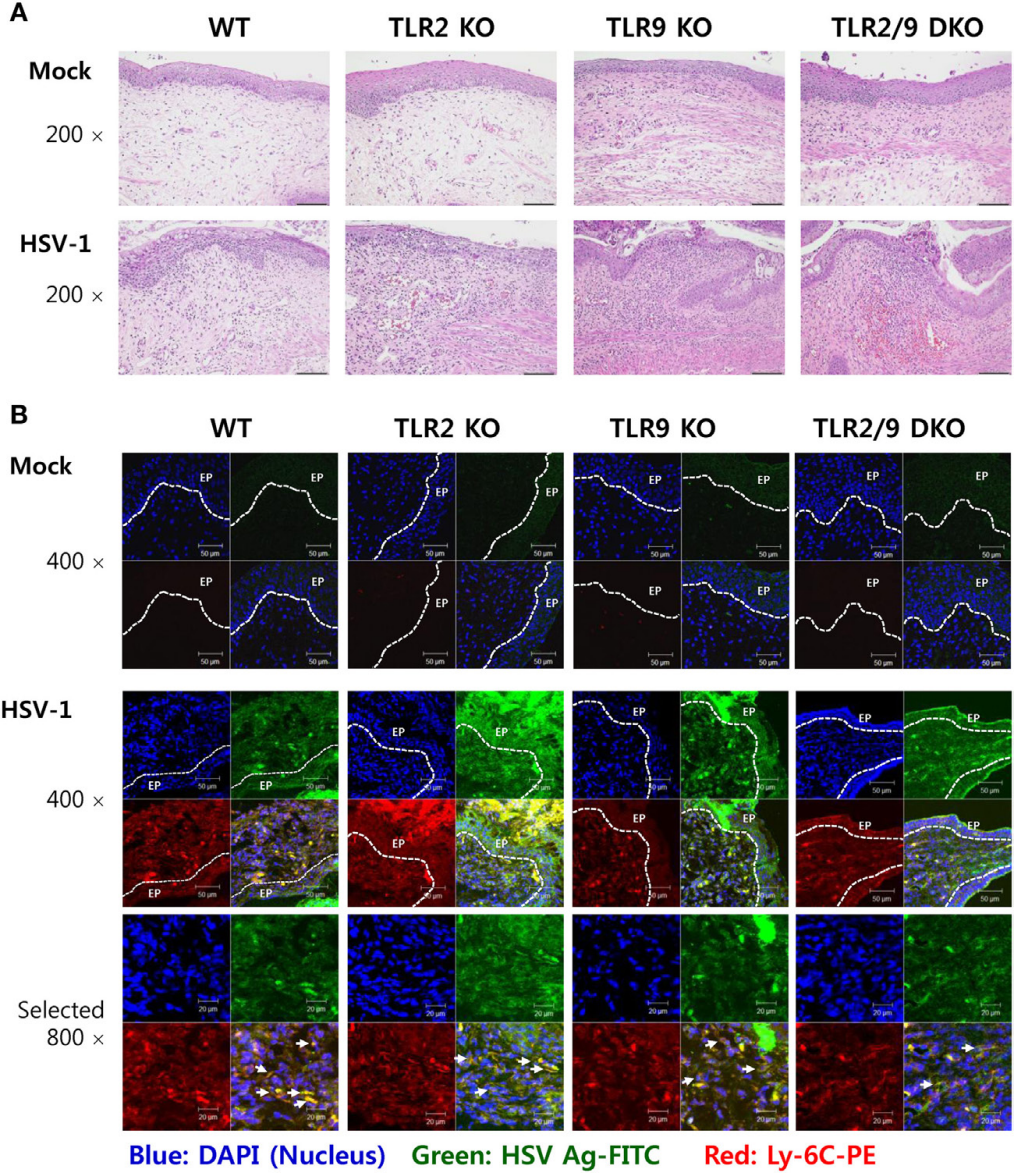
Histopathological examination and confocal microscopy confirm the early accumulation of Ly-6C^+^ monocytes in vaginal tissues following herpes simplex virus (HSV)-1 infection. **(A)** Representative photomicrographs of vaginal tissue sections stained with H&E. Photomicrographs were taken from submucosal and epithelial areas 2 days after mucosal HSV-1 infection (1 × 10^7^ pfu/mouse). Images are representative of sections (200×) from at least four mice. **(B)** Confocal microscopy of vaginal tissues. Sections of vaginal tissues obtained from HSV-infected wild-type (WT) (C57BL/6), TLR2 KO, TLR9 KO, and TLR2/9 double knock-out (TLR2/9 DKO) mice were co-stained for HSV Ag (gB) (green), the nuclear stain DAPI (blue), and the monocyte marker Ly-6C (red) 2 days post-infection. Images are representative of sections (400×) from at least four mice, and the lower image is a selected area magnified by twofold (800×). Some Ly-6C^+^ monocytes co-localized with HSV Ag are denoted by white arrows. White dot-line shows epithelial layer (EP layer). Sections of vaginal tissues obtained from uninfected mice were used for negative control group.

Inflammatory Ly-6C^hi^ monocytes differentiate in the BM and egress into the blood stream. Upon viral infection, Ly-6C^hi^ monocytes enter inflamed tissues from the blood stream in a CCR2-mediated manner, and some of the infiltrated Ly-6C^hi^ monocytes further differentiate into inflammatory DCs, which participate in innate and adaptive immunity (30–32). Infiltrated Ly-6C^hi^ monocytes were also reported to play a role in conferring resistance against mucosal HSV-1 infection through driving Th1-biased CD4^+^ T-cell responses (32). Therefore, we examined recruited myeloid-derived leukocyte subpopulations including Ly-6C^hi^ monocytes in the VT, its draining LNs (ILNs), and SC of infected WT, TLR2 KO, TLR9 KO, and TLR2/9 DKO mice. Our data revealed that both TLR2 and TLR9 were critical for recruitment of CD11b^+^Ly-6C^hi^ monocytes in the VT, ILNs, and SC during the early phase of mucosal HSV-1 infection (Figure 2). WT mice showed early infiltration of Ly-6C^hi^ monocytes with a 3- to 10-fold increase in the VT 2 dpi, compared with Ly-6C^hi^ monocyte levels in TLR2 KO, TLR9 KO, and TLR2/9 DKO mice (Figures 2A,B). Notably, TLR2/9 DKO mice contained the lowest number of Ly-6C^hi^ monocytes with one-tenth of the level in the VT of WT mice. Single ablation of TLR9 resulted in more reduced infiltration of Ly-6C^hi^ monocytes in the VT than TLR2 ablation. By contrast, TLR2/9 DKO mice were found to contain a greater number of accumulated Ly-6G^hi^ granulocytes including Ly-6C^-^Ly-6G^hi^ and Ly-6C^hi^Ly-6G^hi^ cells in the VT 2 and 5 dpi. These results indicate that TLR2 and TLR9 could be involved in differential infiltration of Ly-6C^hi^ monocytes and Ly-6G^hi^ granulocytes in inflamed tissues, depending on disease progression. Likewise, TLR2/9 DKO mice showed highly decreased infiltration of Ly-6C^hi^ monocytes but increased Ly-6G^hi^ granulocyte infiltration in ILNs 2 and 5 dpi (Figures 2C,D). The SC of TLR2/9 DKO mice also displayed infiltration of reduced Ly-6C^hi^ monocytes and increased Ly-6G^hi^ granulocytes with a delayed pattern, as compared with vaginal tissue and ILNs (Figures 2E,F). Histopathological examinations support the severe inflammation in vaginal tissues of TLR2/9 DKO compared with WT, TLR2 KO, and TLR9 KO mice, as shown by the early and enhanced recruitment of inflammatory leukocytes including neutrophils in submucosa area (Figure 3A). TLR9 KO mice showed more significantly extensive inflammation in the vaginal tissues, compared with WT and TLR2 KO mice. By extension, to confirm the reduced infiltration of Ly-6C^hi^ monocytes in the VT of TLR2/9 DKO mice, we examined the localization of Ly-6C^+^ monocytes in vaginal mucosa using confocal microscopy. As expected, infiltrated Ly-6C^+^ monocytes were detected with significantly reduced frequency in the area under the epithelial layer of the VT in TLR2/9 DKO mice compared with WT mice, and some of the Ly-6C^+^ cells co-stained with HSV-1 Ag (Figure 3B). Of note, clustered accumulation of Ly-6C^+^ monocytes was evident in the vaginal submucosa of WT mice, whereas TLR2/9 DKO mice displayed a scattered distribution of Ly-6C^+^ monocytes. Also, TLR9 KO mice showed a more scattered and reduced distribution of Ly-6C^+^ monocytes in the vaginal submucosa than TLR2 KO mice. This implies that TLR9 predominantly functions in the infiltration of Ly-6C^hi^ monocytes during mucosal infection with HSV-1, compared with TLR2. Furthermore, CD11c^+^ DCs and their myeloid DC subpopulation (CD11b^+^CD11c^+^) were detected with lower levels in the VT of TLR2/9 DKO mice than WT, TLR2 KO, and TLR9 KO mice at 2 and 5 dpi (Figure S1A in Supplementary Material). Likewise, ILNs and the SC of TLR2/9 DKO mice contained a reduced number of CD11c^+^ DCs and myeloid CD11b^+^CD11c^+^ DCs compared with WT, TLR2 KO, and TLR9 KO mice (Figures S1B,C in Supplementary Material). Here, of interest, CD11b^+^CD11c^−^ myeloid-derived cells were detected with lower levels in the VT of TLR2/9 DKO mice than in WT, TLR2 KO, and TLR9 KO mice at 2 dpi, but was reversed at 5 dpi (Figure S1A in Supplementary Material). This might be caused by the severe vaginal inflammation of TLR2/9 DKO mice at 5 dpi. Reduced infiltration of CD11b^+^ myeloid-derived cells and CD11c^+^ DCs in the VT of TLR2/9 DKO mice was also confirmed by confocal microscopy at 2 dpi (Figures S1D,E in Supplementary Material). Collectively, these results indicate that TLR2 and TLR9 are essential for the recruitment of Ly-6C^hi^ monocytes and CD11c^+^ DCs in primary inflamed tissue, such as the VT, at the early stage after mucosal infection with HSV-1.

### Dual TLR2/9 Recognition Is Required to Recruit and Activate NK Cells

**Figure 4:**
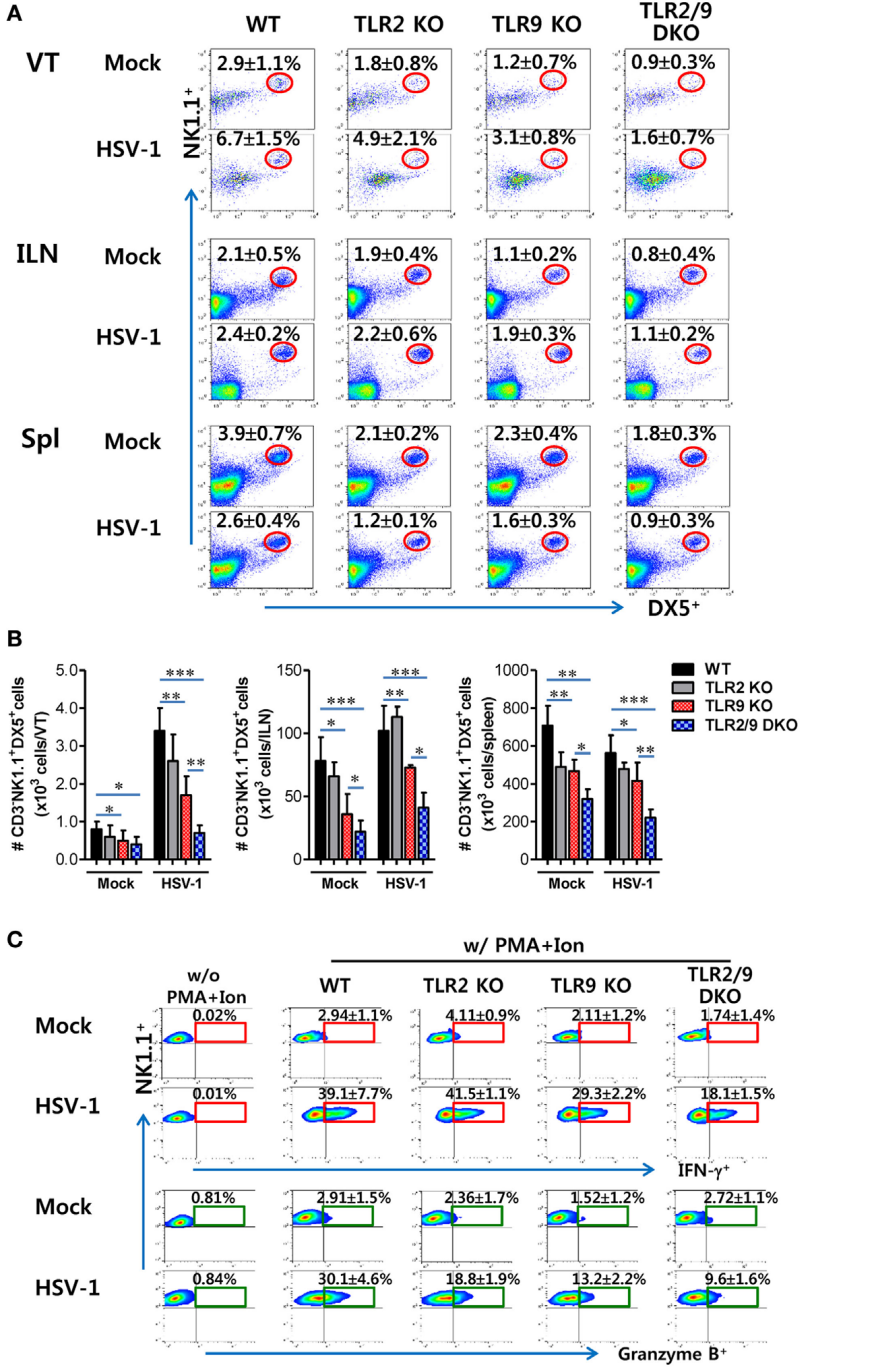

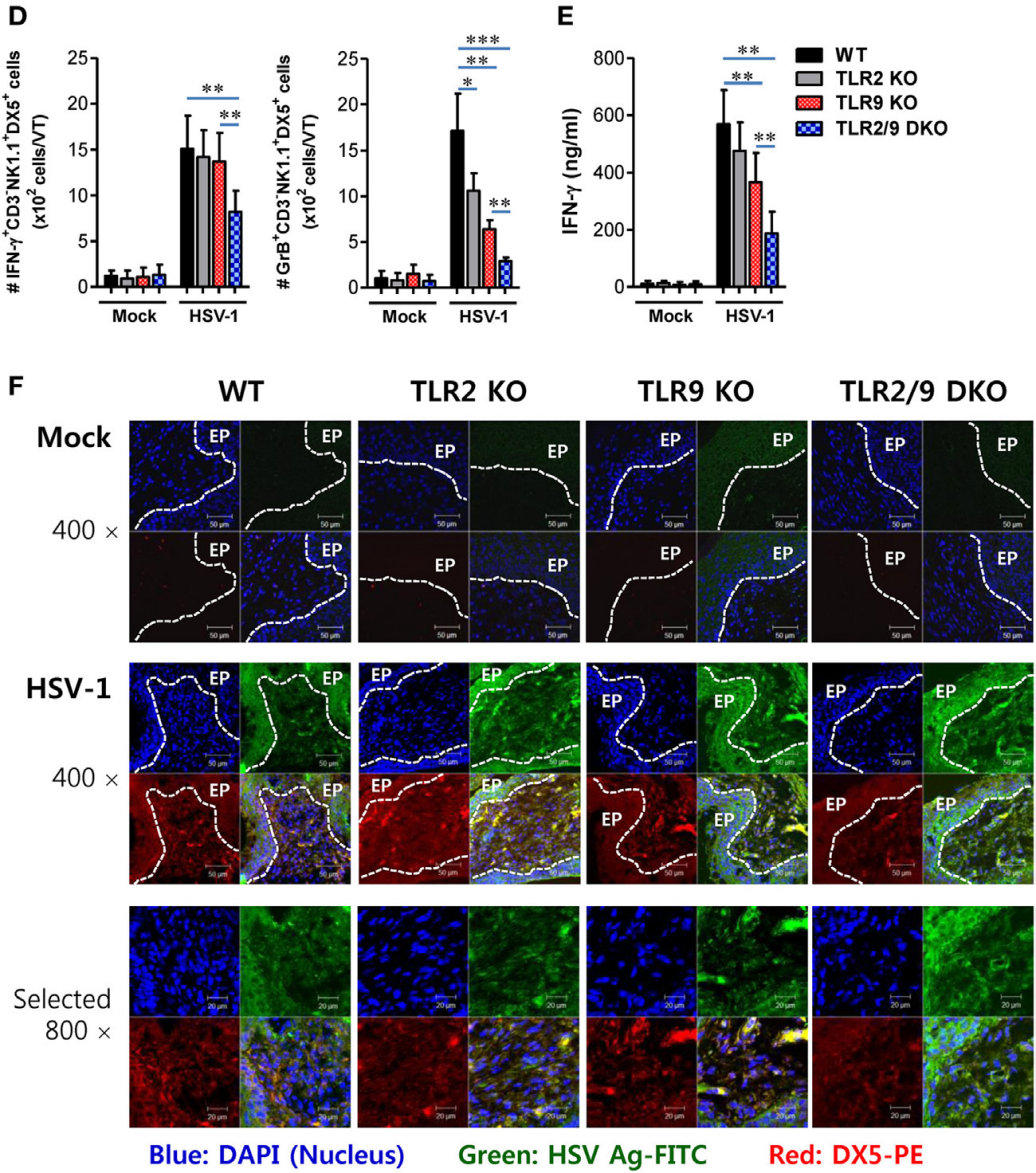
Reduced recruitment and activation of NK cells by the deficiency of TLR2 and TLR9 molecules. **(A)** NK cell infiltration of infected wild-type (WT), TLR2 KO, TLR9 KO, and TLR2/9 double knock-out (TLR2/9 DKO) mice. Cells were prepared from the vaginal tract (VT), iliac LNs (ILNs), and spleen (Spl) with collagenase digestion 0 and 2 days post-infection (dpi), and used for analysis of NK cells. The values in the dot-plots represent the average percentage plus SEM of NK1.1^+^DX5^+^ NK cells after gating on CD3-negative cells (*n* = 4–5). **(B)** Total accumulated NK cell number. The total number of accumulated CD3^−^NK1.1^+^DX5^+^ NK cells in the VT, ILN, and Spl was determined by flow cytometric analysis 2 dpi. **(C)** The frequency of IFN-γ and granzyme B (GrB)-producing cells in vaginal NK cells. The proportion of IFN-γ and GrB-producing cells in CD3^−^NK1.1^+^DX5^+^ NK cells was determined by intracellular staining after brief stimulation of vaginal leukocytes with PMA and ionomycin 0 and 2 dpi. The values in the plots represent the average plus SEM of IFN-γ or GrB-producing cells in NK1.1^+^ cells after gating on CD3^−^NK1.1^+^DX5^+^ NK cells (*n* = 4–5). Vaginal leukocytes unstimulated with PMA and ionomycin were used for negative control. **(D)** The absolute number of IFN-γ or GrB-producing NK cells. The total number of IFN-γ or GrB-producing CD3^−^NK1.1^+^DX5^+^ NK cells was enumerated by flow cytometric analysis using intracellular and surface staining 0 and 2 dpi. **(E)** Secreted IFN-γ levels in vaginal lavages. Secreted IFN-γ levels were determined by ELISA 0 and 2 dpi using vaginal lavages. **(F)** Confocal microscopy of infiltrated NK cells. Sections of vaginal tissues obtained from herpes simplex virus (HSV)-infected mice were co-stained for HSV Ag (gB) (green), the nuclear stain DAPI (blue), and the NK cell marker DX5 (red) 2 dpi. Images are representative of sections (400×) from at least four mice, and the lower image is a selected area magnified by twofold (800×). White dot-line shows epithelial layer (EP layer). Sections of vaginal tissues obtained from uninfected mice were used for negative control group. Two-way ANOVA followed by Bonferroni *post hoc* testing was conducted in *B*. One-way ANOVA with repeated measurements and Bonferroni *post hoc* tests were performed in **(D,E)**. Data in the bar chart represent the average ± SEM of the levels derived from at least three independent experiments (*n* = 3–4). *p < 0.05; **p < 0.01; ***p < 0.001 comparing levels between the indicated groups.

Besides Ly-6C^hi^ monocytes, NK cells are a critical innate cellular component to confer protection against mucosal HSV-1 infection *via* production of IFN-γ and GrB (25, 26). Because vaginal infiltration of Ly-6C^hi^ monocytes was governed by TLR2 and TLR9, we were also interested in testing whether the infiltration and activation of NK cells were affected during mucosal infection with HSV-1. TLR2/9 DKO mice displayed highly decreased infiltration of CD3^-^NK1.1^+^DX5^+^ NK cells in the VT compared with WT, TLR2 KO, and TLR9 KO mice (Figure 4A). NK cells were detected with lower frequency in the VT of TLR9 KO mice than WT and TLR2 KO mice, which indicates that TLR9 may play a more important role in recruiting NK cells than TLR2. Similarly, the ILNs and Spl of TLR2/9 DKO mice contained fewer NK cells compared with those of WT, TLR2 KO, and TLR9 KO mice. Here, one interesting thing was that TLR2/9 DKO and TLR9 KO mice showed basally lower frequency of NK cells in the VT, ILNs, and Spl, as compared with TLR2 KO and WT mice. Supporting these findings, the total accumulated number of CD3^−^NK1.1^+^DX5^+^ NK cells in the VT, ILNs, and Spl was greatly reduced in TLR2/9 DKO mice, and TLR9 KO mice contained a lower total number of NK cells in the three tissues than TLR2 KO mice (Figure 4B). In addition, when the activation of NK cells was examined by analyzing their production of IFN-γ and GrB upon brief stimulation with PMA and ionomycin, TLR2/9 DKO mice showed a greater reduction in IFN-γ and GrB levels in vaginal CD3^−^NK1.1^+^DX5^+^ NK cells (Figure 4C), along with a decrease in the total number of vaginal NK cells producing IFN-γ and GrB compared with WT, TLR2 KO, and TLR9 KO mice (Figure 4D). Also, NK cells detected in VT of TLR2/9 DKO mice showed lower activated phenotypes for CD62L, CD69, and KLRG1 (Figure S2 in Supplementary Material). Consistent with these results, lower levels of IFN-γ protein were detected in vaginal lavages of TLR2/9 DKO mice compared with other animals including WT, TLR2 KO, and TLR9 KO mice (Figure 4E). Furthermore, examination of NK cell recruitment by confocal microscopy supported the reduced infiltration of DX5^+^ NK cells in the VT of TLR2/9 DKO mice (Figure 4F). Clustered accumulation of DX5^+^ NK cells in a subarea of the epithelial dome of the WT VT was evident, whereas TLR2/9 DKO mice showed a scattered appearance of a few DX5^+^ NK cells in the submucosa area of the VT. Similar to the role of TLR2 and TLR9 in vaginal Ly-6C^hi^ monocyte recruitment, these results indicate that vaginal recruitment of NK cells and their activation could be governed by TLR2 and TLR9 during mucosal infection with HSV-1.

### Early Recruitment of Ly-6C^hi^ Monocytes and NK Cells Is Associated With Rapid Responses of CC Chemokines in Mucosal Tissues

**Figure 5:**
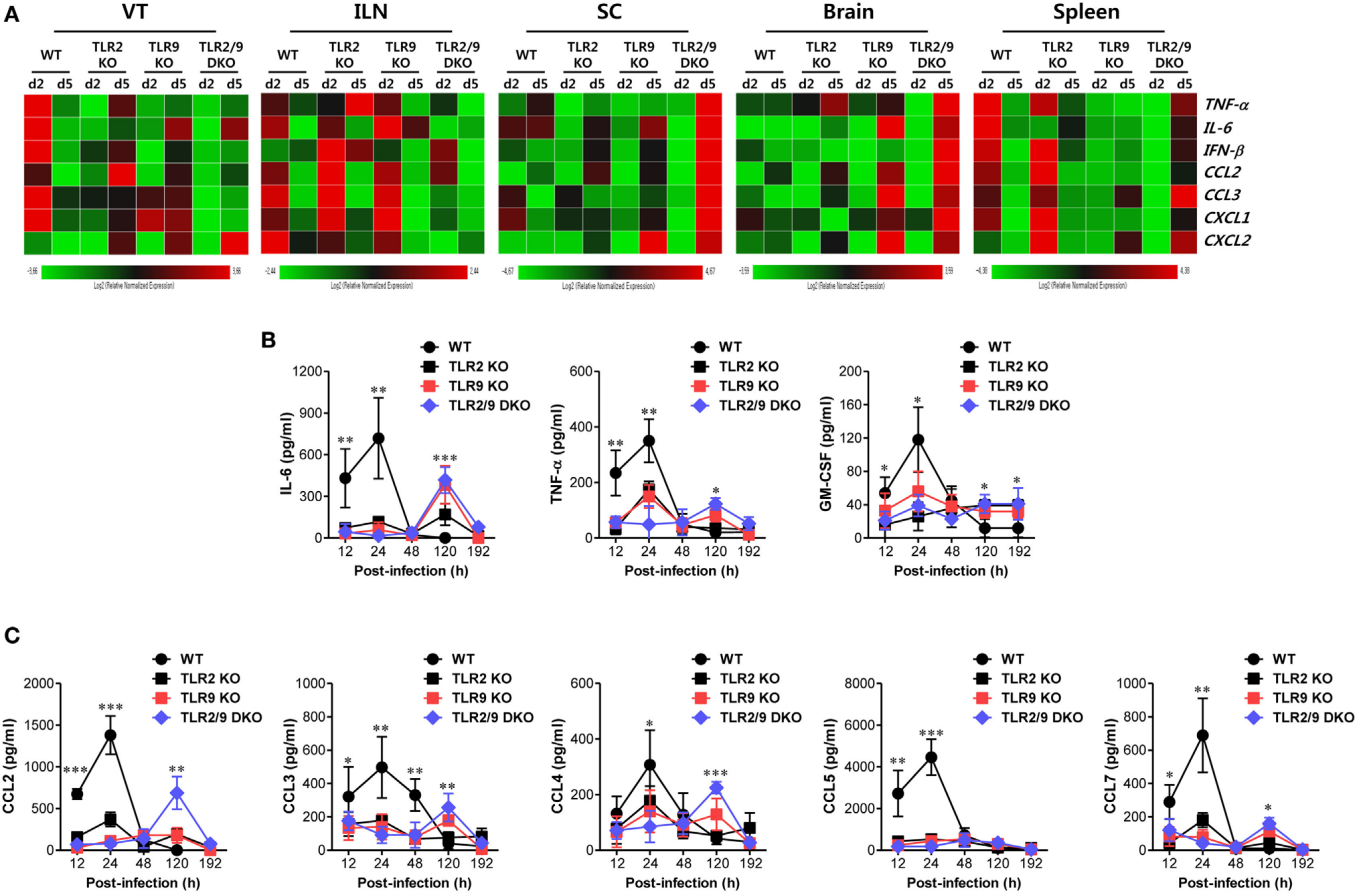
Both TLR2 and TLR9 are required for rapid responses of cytokine and chemokines in vaginal tracts (VTs) infected with herpes simplex virus type 1. **(A)** Heatmap showing the expression of cytokines and chemokines in each tissue. The expression levels of cytokines and chemokines were assessed by real-time qRT-PCR using total RNA extracted from the VT, iliac LNs, spinal cord, brain, and spleen of infected wild-type (WT), TLR2 KO, TLR9 KO, and TLR2/9 double knock-out (TLR2/9 DKO) at 0, 2, and 5 days post-infection (dpi). The expression of each cytokine and chemokine is normalized to the housekeeping gene β-actin and displayed as the average of relative fold expression compared with uninfected control group, according to the indicated color on a log_2_ scale. **(B,C)** Secreted levels of cytokines and chemokines in vaginal lavages. Vaginal lavages were collected at the indicated dpi and used to measure levels of cytokines **(B)** and chemokines **(C)** by CBA. Data show the average ± SEM of values derived from at least three independent experiments (*n* = 4–5). Two-way ANOVA followed by Bonferroni *post hoc* testing was conducted in **(B,C)**. *p < 0.05; **p < 0.01; ***p < 0.001 comparing levels between WT and TLR2/9 DKO mice at the indicated time points.

The complex cascade of responses of cytokines and chemokines provides an orchestrated environment for viral clearance through regulating the recruitment of innate and adaptive immunity-related cellular components (42, 43). Our data demonstrate that both TLR2 and TLR9 are essential to provide antiviral immune responses in the VT *via* early infiltration of Ly-6C^hi^ monocytes and NK cells. Therefore, to further understand antiviral immune responses in the VT of TLR2/9-ablated mice, we examined the expression of cytokines and chemokines in the VT during mucosal infection with HSV-1. TLR2/9 DKO mice showed diminished expression of cytokines and chemokines in the VT, by contrast with the rapid and high expression of cytokines and chemokines in WT mice (Figure 5A). One intriguing result was that CXCL2 expression was greatly increased in the VT of TLR2/9 DKO mice 5 dpi compared with WT, TLR2 KO, and TLR9 KO mice. This might facilitate the recruitment of Ly-6G^hi^ granulocytes in the VT of TLR2/9 DKO mice at a later stage (44). TLR2 KO and TLR9 KO mice showed delayed expression of cytokines and chemokines in the VT 5 days after mucosal infection with HSV-1, as compared with WT mice. ILNs and Spl also showed decreased and delayed patterns of cytokine and chemokine expression in TLR2/9 DKO mice. However, the expression of cytokines and chemokines in CNS tissues including the SC and brain was greatly increased in TLR2/9 DKO mice at a later stage (5 dpi). This higher expression of cytokines and chemokines in CNS tissue of TLR2/9 DKO mice might be caused by increased CNS-invasion of the virus. Supporting these results, WT mice secreted higher amounts of cytokines (IL-6, TNF-α, and GM-CSF) in vaginal lavages at the early stage (2 dpi) compared with TLR2 KO, TLR9 KO, and TLR2/9 DKO mice (Figure 5B). However, TLR2 and/or TLR9-ablated mice contained higher amounts of cytokines in vaginal lavages than WT mice at the later stage (5 dpi), due to severe inflammatory responses. Regarding the secreted chemokine proteins in vaginal lavages, CC chemokines were secreted earlier and at higher levels in WT mice 2 dpi compared with TLR2 KO, TLR9 KO, and TLR2/9 DKO mice (Figure 5C). Of note, WT mice, but not TLR2 and/or TLR9-ablated mice, showed early and greatly increased secretion of CCR2 ligands (CCL2 and CCL7) that are essential receptors for Ly-6C^hi^ monocyte recruitment (45). This production of CCR2 ligands might lead to early recruitment of Ly-6C^hi^ monocytes in the VT of WT mice at the early stage (2 dpi). By contrast, some CC chemokines (CCL2, CCL3, CCL4, and CCL7) were secreted at higher levels in vaginal lavages of TLR2/9 DKO mice at the later stage (5 dpi) as compared with WT mice. These results indicate that TLR2/9 plays a critical role in the cascade of responses of cytokines and chemokines in inflamed tissue after mucosal infection with HSV-1. Also, the stepwise response of chemokines in vaginal tissue of WT mice, but not TLR2 and/or TLR9-ablated mice, was closely associated with the early recruitment of Ly-6C^hi^ monocytes and NK cells.

### TLR2/9 Have a Critical Role in the Differentiation of Ly-6C^hi^ Monocytes Into Tip-DCs

**Figure 6:**
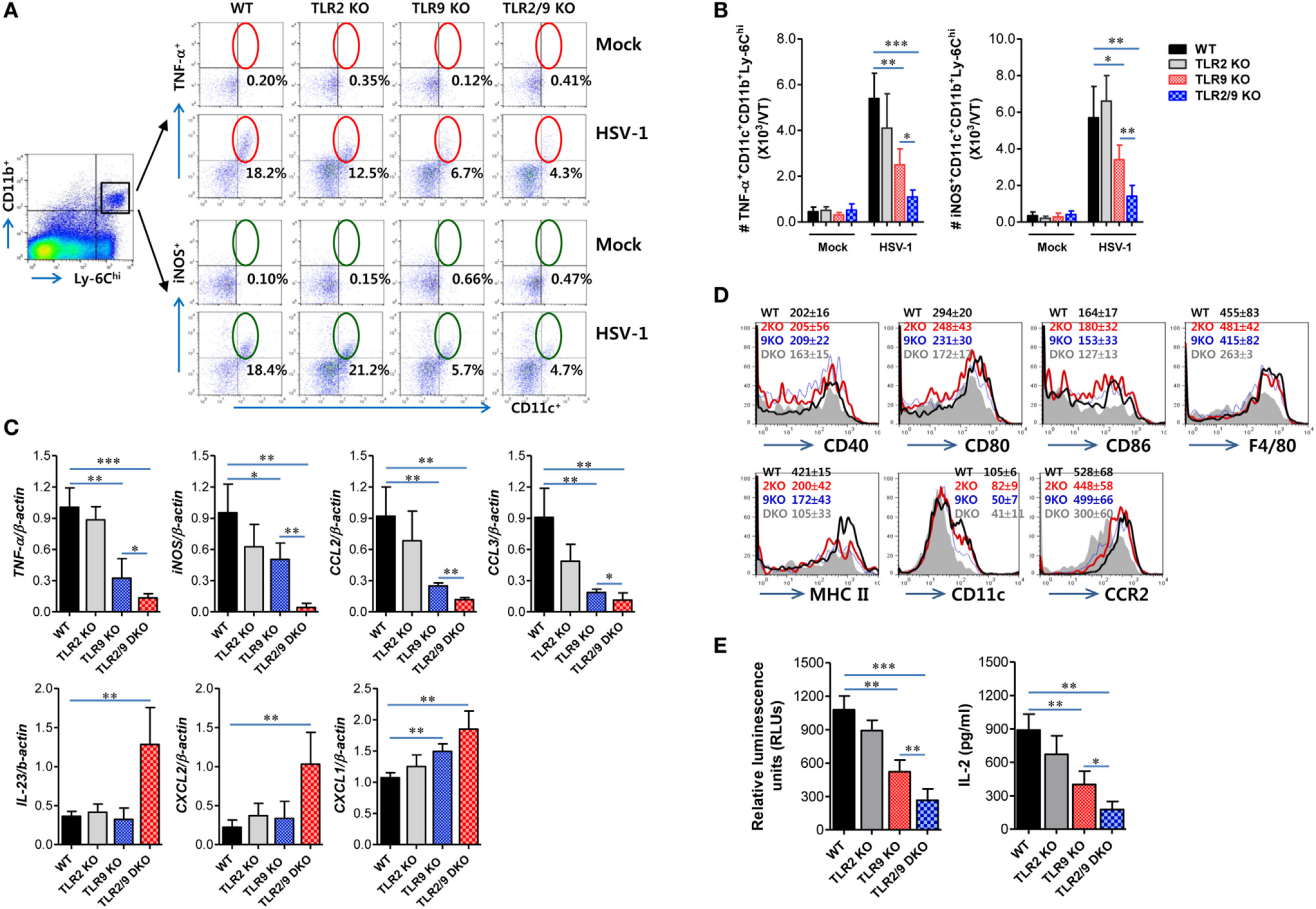
Dual TLR2/9 recognition of herpes simplex virus type 1 to promote the differentiation of Ly-6C^hi^ monocytes in TNF-α^+^iNOS^+^CD11c^+^ TNF-α and iNOS-producing dendritic cells (Tip-DCs). **(A)** The frequency of Tip-DCs in the vaginal tract (VT) of infected mice. Cells were prepared from the VT with collagenase digestion 0 and 2 days post-infection (dpi) and used for analysis of Tip-DCs. Tip-DCs were detected by intracellular TNF-α or iNOS staining combined with surface staining for CD11c, Ly-6C, and CD11b. The values in the dot-plots represent the average percentage of TNF-α^+^ or iNOS^+^CD11c^+^ after gating on CD11b^+^Ly-6C^hi^ cells. **(B)** The total number of accumulated TNF-α^+^ or iNOS^+^CD11c^+^CD11b^+^Ly-6C^hi^ Tip-DCs in the VT. **(C)** The cytokine expression profile of sorted CD11b^+^Ly-6C^hi^ monocytes in the VT. Total RNA was extracted from sorted CD11b^+^Ly-6C^hi^ monocytes in the VTs 2 dpi and used for real-time qRT-PCR. **(D)** Phenotypic levels of vaginal Ly-6C^hi^ monocytes. The values in the histograms denote the average ± SEM of MFI for the indicated surface marker after gating on CD11b^+^Ly-6C^hi^ monocytes. **(E)** Ag-presentation of sorted CD11b^+^Ly-6C^hi^ monocytes. Sorted CD11b^+^Ly-6C^hi^ monocytes in the VTs were co-cultured with OT-II CD4^+^ T cells for 72 h. OT-II CD4^+^ T-cell proliferation was assessed by a viable cell ATP bioluminescence assay, and IL-2 levels in culture supernatants were determined by ELISA. Data in the bar graphs denote the average ± SEM of values derived from at least three independent experiments (*n* = 4–5). Two-way ANOVA followed by Bonferroni *post hoc* testing was conducted in **(B)**. One-way ANOVA with repeated measurements and Bonferroni *post hoc* tests were performed in **(C,E)**. *p < 0.05; **p < 0.01; ***p < 0.001 comparing levels between the indicated groups.

Once inflammatory Ly-6C^hi^ monocytes are recruited in inflamed tissues, they can give rise to MCs including Tip-DC (iNOS^+^ MCs) subset that plays an important role in providing antimicrobial defense (28–32). Therefore, we were interested in exploring whether TLR2/9 could affect the differentiation of Ly-6C^hi^ monocytes into Tip-DCs in inflamed tissues such as the VT after mucosal infection with HSV-1. Ly-6C^hi^ monocytes were observed to give rise to CD11c^+^ DCs producing TNF-α or iNOS in the VT of WT mice with around 18% following mucosal infection with HSV-1, whereas TLR2/9 DKO mice showed drastically reduced differentiation of TNF-α^+^ or iNOS^+^CD11c^+^ Tip-DCs from Ly-6C^hi^ monocytes with levels of around 4% (Figure 6A). Also, TLR9 KO mice showed more impaired differentiation of Tip-DCs from Ly-6C^hi^ monocytes with levels of 5–6%, compared with TLR2 KO mice that showed TNF-α^+^ or iNOS^+^CD11c^+^ DC levels of 12 or 21% in Ly-6C^hi^ monocytes, respectively. This result implies that TLR9 plays a dominant role in differentiating Tip-DCs from Ly-6C^hi^ monocytes during mucosal infection with HSV-1. Consistent with this, TLR2/9 DKO mice contained the lowest number of TNF-α^+^ or iNOS^+^CD11c^+^ Tip-DCs in the VT when the absolute number of Tip-DCs in the VT was determined (Figure 6B). TLR9 KO mice were observed to have a lower number of Tip-DCs in the VT than TLR2 KO mice. Furthermore, we assessed the profile of cytokine expression in vaginal Ly-6C^hi^ monocytes to further clarify Tip-DC features of Ly-6C^hi^ monocytes. As expected, Ly-6C^hi^ monocytes sorted from the VT of WT mice showed high levels of expression of TNF-α and iNOS, whereas Ly-6C^hi^ monocytes derived from TLR2/9 DKO mice expressed TNF-α and iNOS at the lowest levels (Figure 6C). This indicates that Ly-6C^hi^ monocytes derived from WT mice show Tip-DC-like features more than those of TLR2/9 DKO mice. Here, one interesting result was that Ly-6C^hi^ monocytes sorted from the VT of WT mice showed significant expression of CCL2 and CCL3, but Ly-6C^hi^ monocytes derived from TLR2/9 DKO mice showed apparently increased expression of IL-23, CXCL1, and CXCL2. This differential chemokine expression in Ly-6C^hi^ monocytes derived from TLR2/9 DKO mice appeared to facilitate the recruitment of Ly-6G^hi^ granulocytes in the VT. Because Tip-DCs have been known to play a role in presenting Ag to CD4^+^ or CD8^+^ T cells (46), we examined the expression of Ag-presentation-related molecules and some phenotypic markers including F4/80, CCR2, and CD11c. Ly-6C^hi^ monocytes in the VT of TLR2/9 DKO mice showed reduced expression levels of Ag-presentation-related molecules (CD40, CD80, CD86, and MHC II), as compared with Ly-6C^hi^ monocytes in WT, TLR2 KO, and TLR9 KO mice (Figure 6D). In addition, vaginal monocytes of TLR2/9 DKO mice expressed phenotypic markers for DCs with lower levels than those of other animals. Supporting this result, Ly-6C^hi^ monocytes sorted from the VT of TLR2/9 DKO mice showed weak capability of Ag-presentation to OT-II CD4^+^ T cells compared with monocytes sorted from the VT of WT, TLR2 KO, and TLR9 KO mice (Figure 6E). Also, Ly-6C^hi^ monocytes derived from TLR9 KO mice were less able to present Ag than Ly-6C^hi^ monocytes from TLR2 KO mice. Taken together, these results indicate that TLR2/9 plays a critical role in differentiating Tip-DCs from Ly-6C^hi^ monocytes and regulating their functions in the VT during mucosal infection with HSV-1.

### TLR2/9 Recognition Enhances Functional T-Cell Responses in Inflamed Tissues and Draining LNs Following Mucosal HSV-1 Infection

**Figure 7:**
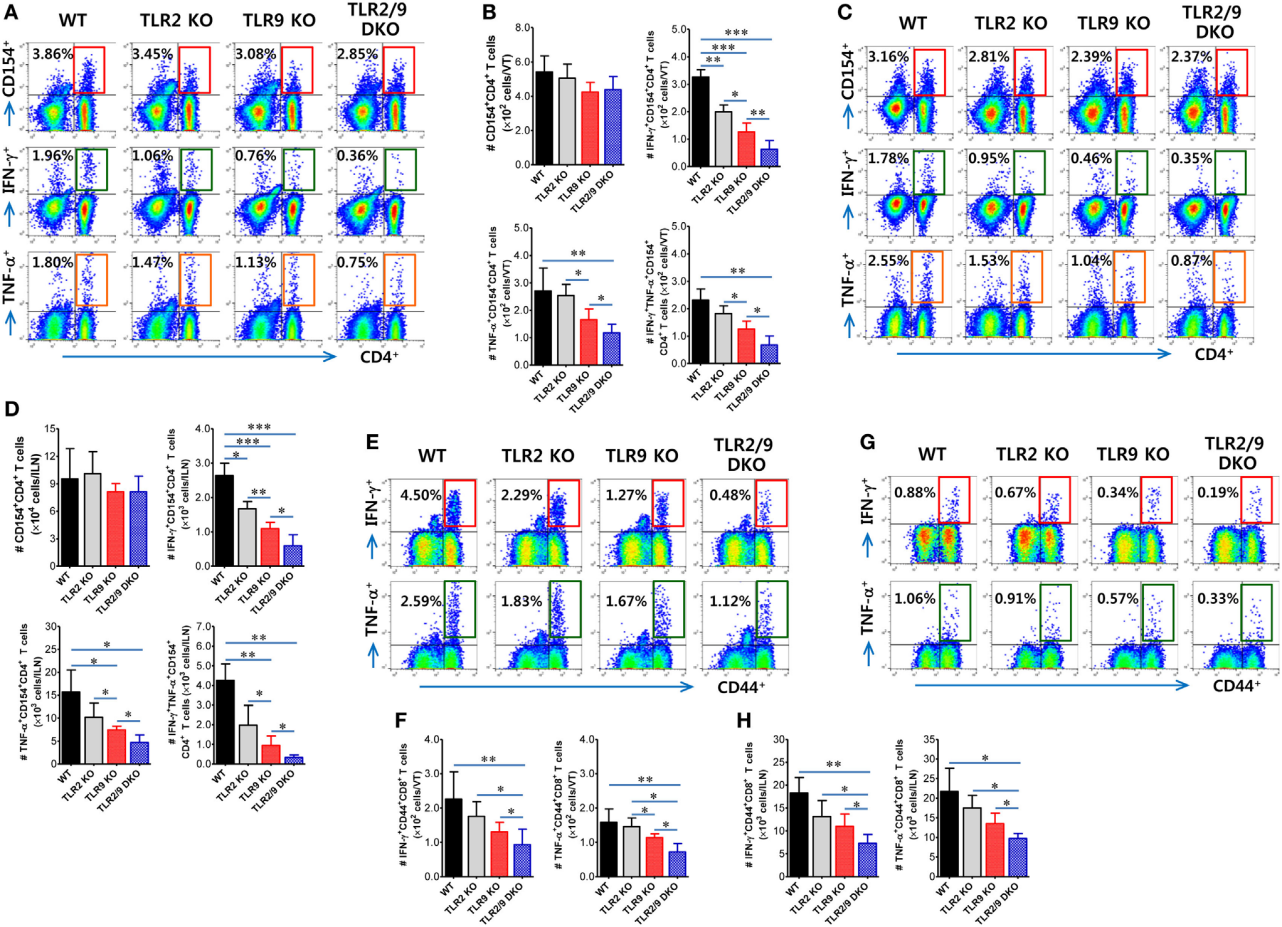
Herpes simplex virus (HSV)-1 Ag-specific CD4^+^ and CD8^+^ T-cell responses in the vaginal tract (VT) and its draining LNs of infected mice. **(A–D)** The frequency and absolute number of CD4^+^ T cells specific for HSV-1 Ag in the VT and iliac LNs (ILNs). **(E–H)** The frequency and absolute number of CD8^+^ T cells specific for the immunodominant epitope (gB_498-505_, SSIEFARL) of HSV-1 in the VT and ILNs. Leukocytes were prepared from the VT (VT) and ILNs of surviving mice 7 days post-infection and co-cultured with UV-inactivated HSV-1 or gB_498-505_ peptide-pulsed APCs for CD4^+^ or CD8^+^ T-cell responses, respectively. HSV-1 Ag-specific CD4^+^ and CD8^+^ T cells were detected by intracellular IFN-γ and TNF-α staining combined with CD4 or CD8 surface staining. The values in the dot-plots represent the average percentage of each population (CD154^+^, IFN-γ^+^, or TNF-α^+^) after gating on CD4^+^ or CD8^+^ cells. Data in the bar graphs denote the average ± SEM of values derived from at least three independent experiments (*n* = 4–5). One-way ANOVA with repeated measurements and Bonferroni *post hoc* tests were performed in **(B,D,F,H)**. *p < 0.05; **p < 0.01; ***p < 0.001 comparing levels between the indicated groups.

TNF-α and iNOS-producing DCs differentiated from Ly-6C^hi^ monocytes migrate from inflamed sites to draining LNs and share functional features of classical DCs that stimulate CD4^+^ and CD8^+^ T-cell responses (47). In this study, dual recognition of HSV-1 infection by TLR2/9 appeared essential to promote the differentiation of Tip-DCs in the VT. Moreover, Tip-DCs in the ILNs of WT mice were detected with increased levels compared with other animals (data not shown). Therefore, we were interested in exploring the *in vivo* capacity of TLR2 and/or TLR9-deficient Tip-DCs to stimulate Ag-specific CD4^+^ and CD8^+^ T-cell responses in the VT and its draining LNs. To this end, we assessed HSV-1 Ag-specific CD4^+^ and CD8^+^ T-cell responses in the VT and ILNs 7 days after mucosal infection with HSV-1. Although this analysis could not exclude the contribution of classical DCs in the draining LNs of the VT, the comparable possibility of TLR2 and/or TLR9-deficient Tip-DCs facilitating T-cell responses could be evaluated. In strong support of the impaired maturation of Tip-DCs in TLR2/9 DKO mice, TLR2/9 DKO mice showed a drastically reduced frequency of IFN-γ or TNF-α-producing HSV-1-specific CD4^+^ T cells in the VT in response to stimulation with UV-inactivated HSV-1 (Figure 7A). However, TLR2/9 DKO mice contained a comparable number of vaginal HSV-1-specific CD4^+^ T cells as WT mice based on the enumeration of HSV-1-specific CD4^+^ T cells by intracellular CD154 staining (38). This result implies that impaired Tip-DC maturation in TLR2/9 DKO mice may be involved in the poor expansion of functional cytokine-producing effector CD4^+^ T cells. Also, a comparable number of HSV-1-specific CD154^+^CD4^+^ T cells in the VT of WT, TLR2 KO, TLR9 KO, and TLR2/9 DKO mice was detected when the total number of HSV-1-specific CD4^+^ T cells was enumerated, whereas TLR2/9 DKO mice contained a highly reduced number of IFN-γ and/or TNF-α-producing CD154^+^CD4^+^ T cells compared with other animals (Figure 7B). TLR9 KO mice had a lower number of cytokine-producing CD154^+^CD4^+^ T cells in the VT than TLR2 KO mice. Likewise, TLR2/9 DKO mice showed the lowest responses of HSV-1-specific CD4^+^ T cells in the draining LNs (ILNs) (Figures 7C,D). Of note, TLR2/9 DKO mice contained a much lower total number of functional cytokine-producing CD154^+^CD4^+^ T cells in the ILNs with one-tenth of the levels observed in WT mice. Consistent with HSV-1-specific CD4^+^ T-cell responses, IFN-γ-producing CD8^+^ T cells specific for the immunodominant epitope (gB_498-505_, SSIEFARL) of HSV-1 were detected in the VT of TLR2/9 DKO mice with 10-fold-reduced levels in frequency compared with WT mice (Figure 7E). Also, the lowest number of TNF-α-producing CD8^+^ T cells was found in the VT of TLR2/9 DKO mice. Supportively, TLR2/9 DKO mice were observed to contain the lowest number of gB_498-505_-specific CD8^+^ T cells in the VT when examined for IFN-γ or TNF-α-producing CD8^+^ T cells in response to stimulation with an epitope peptide (Figure 7F). ILNs of TLR2/9 DKO mice also contained a lower frequency and total number of gB_498-505_-specific CD8^+^ T cells than WT mice as well as TLR2 KO and TLR9 KO mice (Figures 7G,H). Therefore, these results indicate that TLR2/9 molecules play a critical role in generating functional effector CD4^+^ and CD8^+^ T-cell responses against HSV-1 Ag in inflamed tissues and their draining LNs.

### Direct TLR2/9 Recognition Is Required for Ly-6C^hi^ Monocyte and NK Cell Activation Upon HSV-1 Infection

**Figure 8:**
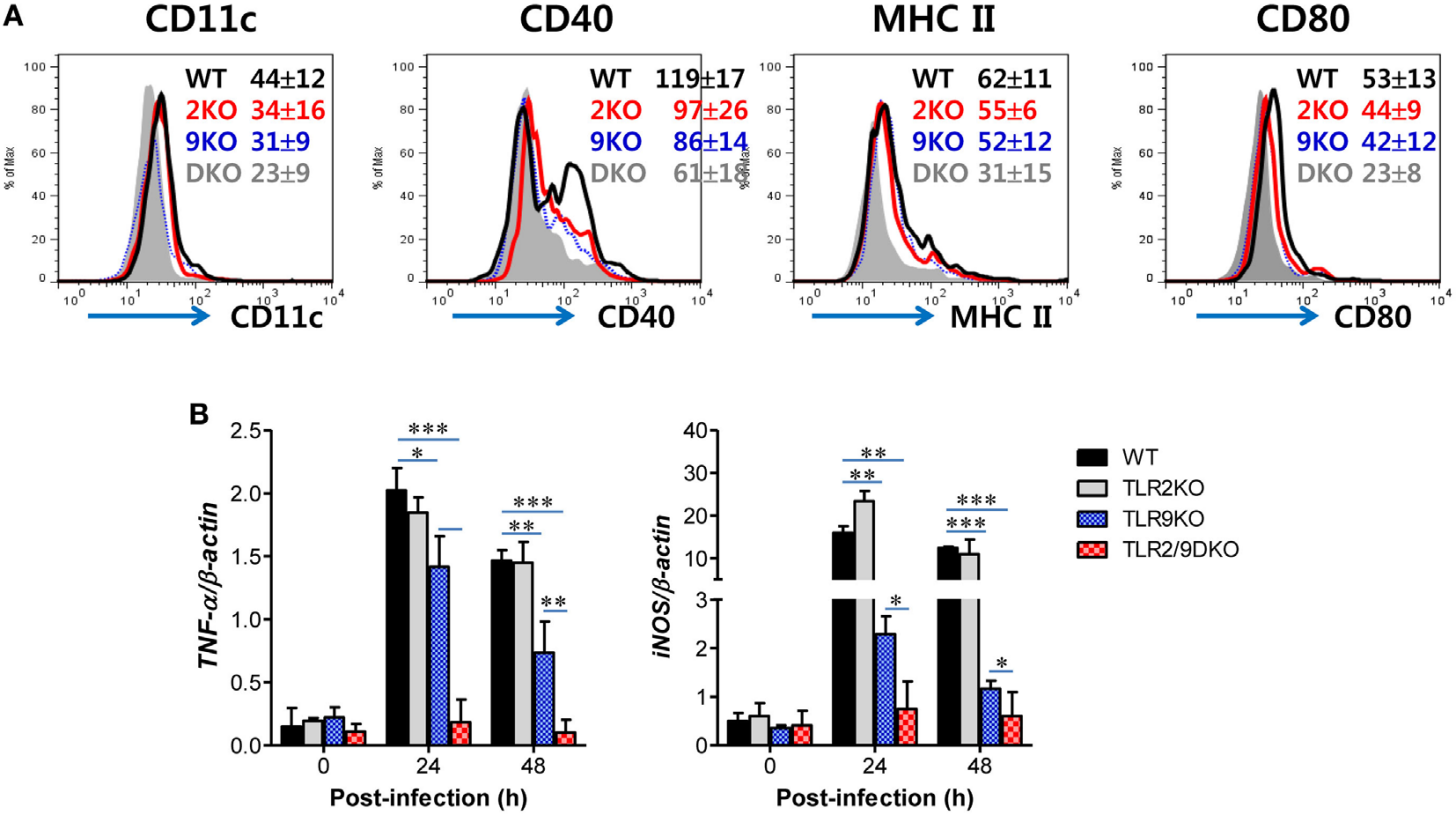
Direct recognition of both TLR2 and TLR9 to promote Ly-6C^hi^ monocyte differentiation into TNF-α and iNOS-producing dendritic cells. **(A)** Phenotypic changes of sorted Ly-6C^hi^ monocytes after herpes simplex virus (HSV)-1 infection. Ly-6C^hi^ monocytes sorted from the spleen of wild-type (WT), TLR2 KO, TLR9 KO, and TLR2/9 double knock-out (TLR2/9 DKO) mice were infected with HSV-1 (5.0 moi) and used for surface staining with the indicated markers 24 h later. The values in the histograms denote the average ± SEM of MFI for the indicated surface marker after gating on CD11b^+^Ly-6C^hi^ monocytes. **(B)** TNF-α and iNOS expression in HSV-1-infected Ly-6C^hi^ monocytes. The levels of TNF-α and iNOS mRNA were determined by real-time qRT-PCR using the total RNA extracted from sorted Ly-6C^hi^ monocytes 24 h after HSV-1 infection. Data in the bar graphs denote the average ± SEM of values derived from at least three independent experiments (*n* = 4–5). Two-way ANOVA followed by Bonferroni *post hoc* testing was conducted in **(B)**. *p < 0.05; **p < 0.01; ***p < 0.001 comparing levels between the indicated groups.

The conversion of Ly-6C^hi^ monocytes to Tip-DCs can be accomplished by stimulation with several soluble factors including IL-12 and IFN-γ that are produced during inflammation (48). Moreover, MyD88 has been reported to be required for the maturation of functional Tip-DCs from Ly-6C^hi^ monocytes (35), which suggests that functional maturation of Tip-DCs could be facilitated by TLR recognition of viral infection. Based on these findings, we next tested the intrinsic role of TLR2/9 in maturation of Tip-DCs from Ly-6C^hi^ monocytes since Ly-6C^hi^ monocytes expressed a wide range of TLRs including TLR2 and TLR9 at various levels (Figure S3A in Supplementary Material). Our results revealed that purified Ly-6C^hi^ monocytes showed up-regulated expression of Ag-presentation-related molecules (CD40, MHC II, and CD80) and a DC marker (CD11c) after HSV-1 infection (Figure 8A). Of note, Ly-6C^hi^ monocytes purified from WT mice displayed greatly increased expression of Ag-presentation-related molecules and CD11c after HSV-1 infection, as compared with Ly-6C^hi^ monocytes purified from TLR2/9 DKO mice. Ly-6C^hi^ monocytes purified from TLR2 KO mice showed slightly higher expression of Ag-presentation-related molecules and CD11c than Ly-6C^hi^ monocytes derived from TLR9 KO mice. Furthermore, we examined the expression of TNF-α and iNOS as functional factors of Tip-DCs in purified Ly-6C^hi^ monocytes after HSV-1 infection. Greatly increased levels of TNF-α and iNOS expression were induced in Ly-6C^hi^ monocytes purified from WT mice, as compared with Ly-6C^hi^ monocytes purified from TLR2/9 DKO mice (Figure 8B). Also, Ly-6C^hi^ monocytes purified from TLR2 KO and TLR9 KO mice showed a significant induction of TNF-α and iNOS expression with HSV-1 infection, but TLR9-deficient monocytes showed lower induction of TNF-α and iNOS expression than monocytes from TLR2 KO mice. These results indicate that dual recognition of TLR2 and TLR9 for HSV-1 infection could directly lead to up-regulation of the functional features of Tip-DCs from Ly-6C^hi^ monocytes.

**Figure 9:**
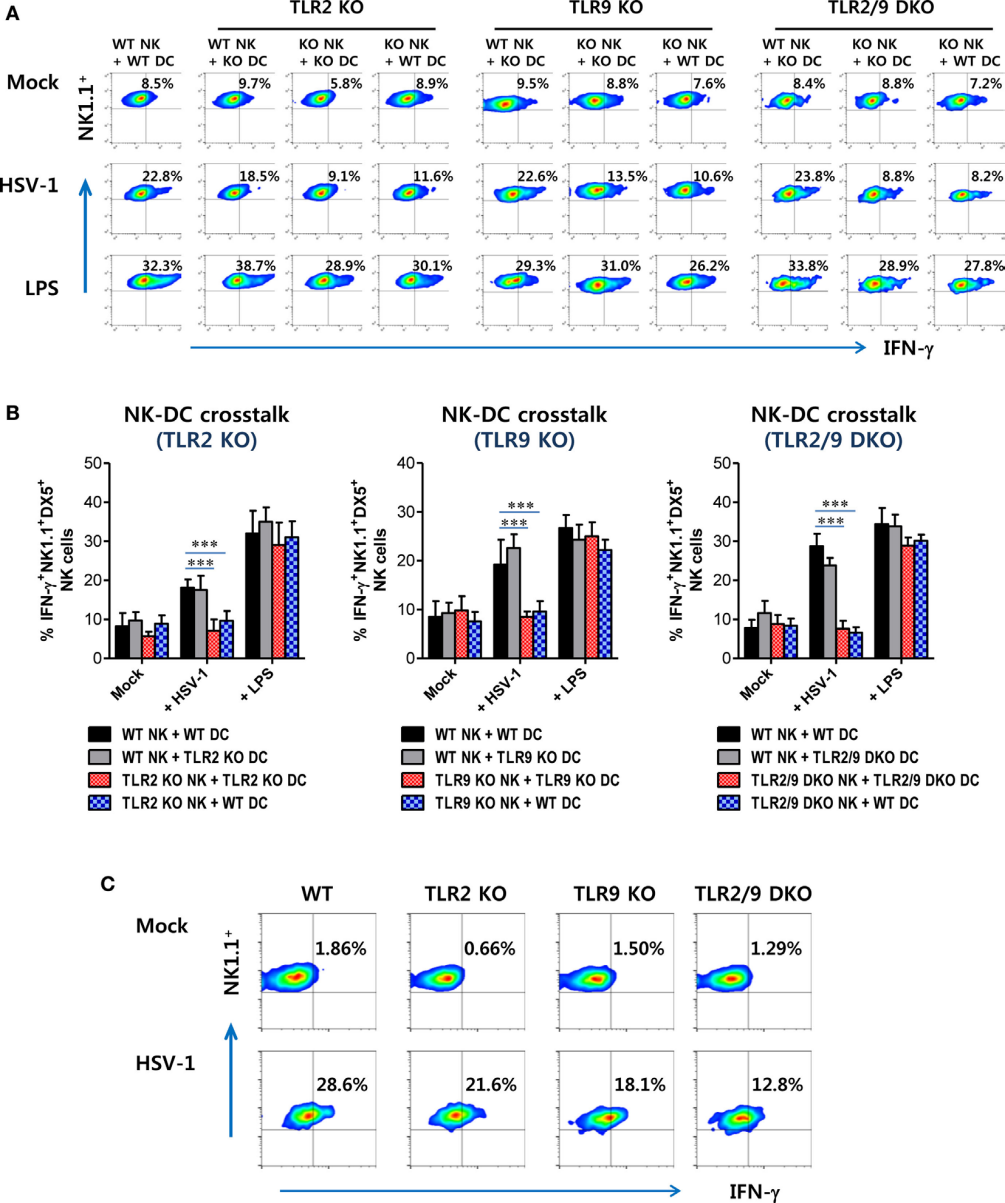
Both TLR2 and TLR9 signaling on NK cells, but not dendritic cells (DCs), are required for NK cell activation upon herpes simplex virus (HSV)-1 infection. Sorted CD3^-^NK1.1^+^DX5^+^ NK cells from the spleen (Spl) of wild-type (WT), TLR2 KO, TLR9 KO, and TLR2/9 double knock-out (TLR2/9 DKO) mice were co-cultured with CD11c^+^ DCs derived from bone marrow cells of WT, TLR2 KO, TLR9 KO, or TLR2/9 DKO mice and stimulated with HSV-1, lipopolysaccharide (LPS), or left uninfected (Mock). The activity of NK cells was assayed by intracellular IFN-γ staining 48 h after infection. **(A)** The plots that show the percentage of IFN-γ-positive cells among CD3^-^NK1.1^+^DX5^+^ NK cells. **(B)** The mean percentage ± SEM of IFN-γ-positive cells among CD3^-^NK1.1^+^DX5^+^ NK cells. **(C)** Direct activation of NK cells by dual TLR2/9 recognition. Sorted NK cells from the Spl of WT, TLR2 KO, TLR9 KO, and TLR2/9 DKO mice were infected with HSV-1 (5.0 moi) and assayed for intracellular IFN-γ 24 h later. The values in the plots represent the average percentage of IFN-γ-producing cells in NK1.1^+^ cells after gating on CD3^-^NK1.1^+^DX5^+^ NK cells. One-way ANOVA with repeated measurements and Bonferroni *post hoc* tests were performed in **(B)**. Data shown are representative of three independent experiments (*n* = 4–5). ***p < 0.001 comparing levels between the indicated groups.

Furthermore, NK cell activation can also be accomplished by direct TLR2 and TLR9 recognition, because CD3^-^NK1.1^+^DX5^+^ NK cells express multiple TLRs including TLR2 and TLR9 (Figure S3B in Supplementary Material). NK cell activation has been shown to be regulated by DC-derived cytokines (IFN-I, IL-12, and IL-15) and/or cell-to-cell contact between DCs and NK cells (49–51). DCs are believed to recognize HSV-1 infection *via* TLR2 and TLR9, and then produce cytokines that are involved in NK cell activation (49–51). To rule out the role of TLR2 and TLR9 signaling on accessory cells including DCs in NK cell activation, we utilized an *in vitro* DC–NK cell co-culture system. CD3^-^NK1.1^+^DX5^+^ NK cells purified from WT, TLR2 KO, TLR9 KO, or TLR2/9 DKO mice were co-cultured *in vitro* with WT, TLR2, TLR9, or TLR2/9-deficient DCs prepared from BM cells in the absence or presence of HSV-1 infection. LPS treatment was used for a positive control. Our data showed that similar amounts of IFN-γ were produced by WT NK cells co-cultured with TLR2, TLR9, or TLR2/9-deficient DCs in the presence of HSV-1 infection, as compared with WT NK cells co-cultured with WT DCs (Figures 9A,B). However, NK cells derived from TLR2 KO, TLR9 KO, and TLR2/9 DKO mice showed highly reduced production of IFN-γ when co-cultured with DCs derived from WT, TLR2 KO, TLR9 KO, and TLR2/9 DKO mice in the presence of HSV-1 infection. This result indicates that NK cell activation is independent of TLR2 and TLR9 signaling on DCs in response to HSV-1 infection. Instead, direct signaling of TLR2 and TLR9 on NK cells appeared to play a critical role in activating NK cells. Also, the lack of IFN-γ production by NK cells derived from TLR2 KO, TLR9 KO, or TLR2/9 DKO mice was unlikely to be driven by their inherent inability to be activated, as TLR2, TLR9, or TLR2/9-deficient NK cells stimulated with the TLR4 ligand, LPS, produced comparable amounts of IFN-γ to WT NK cells (Figures 9A,B). To further support the role of direct TLR2 and TLR9 signaling on NK cell activation, purified and accessory cell-free NK cells were stimulated with HSV-1 and assayed for NK cell activation 48 h later. HSV-1 infection stimulated NK cells to significantly produce IFN-γ, and NK cells purified from TLR2/9 DKO mice showed the lowest production of IFN-γ by HSV-1 infection (Figure 9C). Taken together, these results suggest that functional activation of Ly-6C^hi^ monocytes and NK cells could be directly facilitated by dual recognition of TLR2/9 expressed in corresponding cells during mucosal infection with HSV-1.

### Activation of the p38 MAPK Pathway in NK Cell Activation by Dual TLR2/9 Recognition of HSV-1

**Figure 10:**
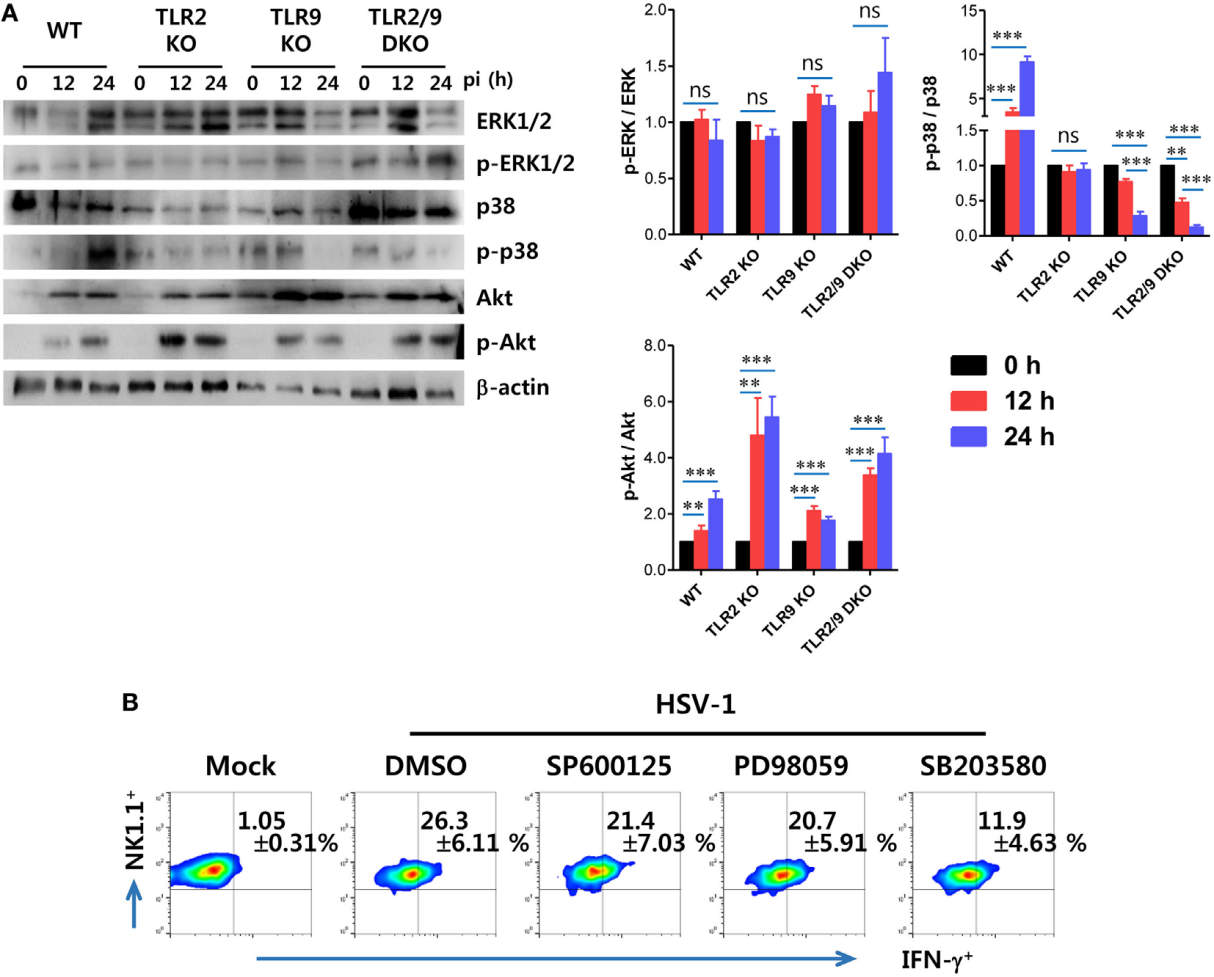
Direct TLR2/9-dependent activation of NK cells by herpes simplex virus (HSV)-1 is mediated by p38 MAPK. **(A)** Phosphorylation of MAPKs in HSV-1 infected NK cells. NK cells purified from the spleen (Spl) of wild-type (WT), TLR2 KO, TLR9 KO, and TLR2/9 double knock-out (TLR2/9 DKO) mice were infected with HSV-1 (5.0 moi). Western immunoblot was used for detecting unphosphorylated and phosphorylated forms of target MAPKs using specific Abs at the indicated time points. The luminescence intensities of Western blot bands were expressed by relative fold intensity compared with uninfected NK cells. **(B)** Dependence of HSV-1-mediated NK cell activation on p38 MAPK. NK cells purified from the Spl of WT mice were infected with HSV-1 in the presence or absence of a JNK inhibitor (SP600125), MEK inhibitor (PD98059), and p38 inhibitor (SB203580). NK cell activation was evaluated by intracellular IFN-γ staining 24 h later. The values in the plots represent the percentage of IFN-γ-producing cells in NK1.1^+^ cells after gating on CD3^-^NK1.1^+^DX5^+^ NK cells. Data in bar graphs and the plots denote the average ± SEM of values derived from at least three independent experiments (*n* = 4–5). Two-way ANOVA followed by Bonferroni *post hoc* testing was conducted for the comparison of luminescence intensities in **(B)**. Data shown are representative of three independent experiments (*n* = 4–5).

TLR2 is believed to be located on cell surface, whereas TLR9 is expressed within endosomes (7–10). Although they have different subcellular locations, TLR2 and TLR9 use the same adaptor molecule, MyD88, to transmit signals to a cascade of MAP kinases including ERK, JNK, and p38 that ultimately lead to the activation of transcription of inflammatory cytokines (52). Next, we examined that which MAPK pathways were activated by stimulation of TLR2/9-MyD88 signaling in NK cell activation by HSV-1. To this end, we measured the phosphorylation of ERK, p38, and Akt MAPKs 0, 12, and 24 h after HSV-1 infection, using NK cells purified from WT, TLR2 KO, TLR9 KO, and TLR2/9 DKO mice. The phosphorylation of ERK in NK cells purified from WT, TLR2 KO, TLR9 KO, and TLR2/9 DKO mice was not apparently observed, except that NK cells derived from TLR2/9 DKO mice showed slightly increased phosphorylation of ERK at 24 hpi (Figure 10A). However, somewhat interestingly, HSV-1 infection induced phosphorylation of Akt MAPK in NK cells derived from WT, TLR2 KO, TLR9 KO, and TLR2/9 DKO mice, whereas the phosphorylation of p38 MAPK was observed in NK cells purified from only WT mice except TLR2 KO, TLR9 KO, and TLR2/9 DKO mice. Instead, NK cells obtained from TLR2/9 DKO mice showed specifically and drastically reduced phosphorylation of p38 MAPK at both 12 and 24 hpi, as compared with WT and TLR2 KO mice. The reduction of p38 phosphorylation in NK cells purified from TLR9 KO mice was observed with delayed pattern at 24 hpi, as compared with TLR2/9 DKO mice. This finding implies that NK cell activation by dual TLR2/9 recognition of HSV-1 infection is mediated by the p38 MAPK pathway. To further confirm the role of p38 MAPK in NK cell activation through TLR2/9 signaling, we assessed NK cell activation after HSV-1 infection in the presence of MAPK inhibitors such as a JNK inhibitor (SP600125), MEK inhibitor (PD98059), and p38 inhibitor (SB203580). NK cell activation by HSV-1 infection was significantly reduced after treatment with a p38 inhibitor (SB203580), as compared with treatment with JNK inhibitor (SP600125) and MEK inhibitor (PD98059) (Figure 10B). Therefore, these results indicate that direct NK cell activation by dual TLR2/9 recognition of HSV-1 is mediated by activation of the p38 MAPK pathway triggered by MyD88-TRAF6 activation.

## Discussion

CD11b^+^Ly-6C^hi^ monocytes egress massively from the BM into the bloodstream in a CCR2-dependent manner upon pathogenic infection, and are then recruited into inflamed tissues *via* the CCR2-CCL2 axis. There, host-derived inflammatory cytokines and chemokines or pathogen-derived molecules activate the infiltrated Ly-6C^hi^ monocytes to upregulate CD11c, CD80/86, and MHC class II, thereby facilitating their maturation toward monocyte-derived DCs including Tip-DCs (28–32). Bosschaert et al. demonstrated that the conversion of Ly-6C^hi^ monocytes to Tip-DCs consists of a three-step process including (i) a CCR2-depedent step crucial for emigration of Ly-6C^hi^ monocytes from the BM followed by (ii) a differentiation step of infiltrated Ly-6C^hi^ monocytes to immature inflammatory DCs (CD11c^+^ but CD80/CD86/MHC II^lo^) which is IFN-γ and MyD88 signaling-independent and (iii) a maturation step of inflammatory DCs to functional (CD80/CD86/MHC II^hi^) TNF-α and iNOS-producing Tip-DCs which is dependent on IFN-γ and MyD88 signaling (35). This notion indicates that the differentiation of Tip-DCs from Ly-6C^hi^ monocytes could be governed by the microenvironment orchestrated by multiple factors including cytokines and pathogen-derived molecules, i.e., PAMPs, in inflamed tissues. Notably, considering that monocyte-derived DCs in MyD88 KO and IFN-γ KO mice express CD80/86 and MHC II molecule but produce TNF-α and iNOS with low levels (35), MyD88 and IFN-γ signaling may be involved in the maturation of monocyte-derived DCs to functional Tip-DCs. Therefore, TLR2/9 expressed in Ly-6C^hi^ monocytes may contribute to the functional maturation of Tip-DCs with help from IFN-γ produced from NK cells in the VT, even though the role of IFN-γ was not examined in this study. Supporting this speculation, our results showed that HSV-1 infection directly promotes Tip-DC-like features in Ly-6C^hi^ monocytes sorted from WT mice by increasing the expression of CD11c, MHC II, TNF-α, and iNOS, but TLR2 and/or TLR9-deficient Ly-6C^hi^ monocytes did not show such phenomena. The currently identified TLR2/9 ligands of HSV-1, gH/L and gB as well as CpG DNA, could represent candidates that contribute to the functional maturation of monocyte-derived DCs to Tip-DCs (7, 8). However, TLR2/9 and IFN-γ are not the only factors thought to be involved in Tip-DC maturation. Other factors produced in inflamed tissues, such as GM-CSF, IL-12p40, and TNF-α, may act in concert with TLR2/9 and IFN-γ to induce the differentiation of Tip-DCs. In support of this idea, WT mice expressed such factors in the VT with highly increased levels at the early stage after HSV-1 infection compared with TLR2 and/or TLR9 KO mice. Although a dissected investigation of the multiple factors produced in the VT is needed to uncover their comparable contribution to the differentiation and maturation of Tip-DCs from Ly-6C^hi^ monocytes, our results revealed one aspect that TLR2/9 can directly contribute to the facilitation of Tip-DC-like features from Ly-6C^hi^ monocytes.

TNF-α and iNOS-producing DCs share some functional features with classical DCs that are differentiated from common DC precursor-derived from hematopoietic stem cells (29). Classical CD11c^+^ DCs are well known to initiate T-cell responses and restimulate effector and memory T-cell responses (53). The Iwasaki group demonstrated that classical and tissue-resident CD11c^+^ DCs are primarily involved in priming of naive CD4^+^ T cells (53), whereas Ly-6C^hi^ monocyte-derived DCs regulate Th1-type cytokine production from effector CD4^+^ T cells (32). This suggests that Ly-6C^hi^ monocyte-derived Tip-DCs are dedicated to maintaining function of effector T cells in inflamed tissues. Their findings strongly strengthen our results that TLR2/9 DKO mice contained a comparable number of vaginal HSV-1-specific CD154^+^CD4^+^ T cells as WT mice (38), while TLR2/9 DKO mice showed a greatly reduced number of functional IFN-γ and/or TNF-α-producing cells in CD154^+^CD4^+^ T cells. Although the intrinsic ability of classical TLR2 and/or TLR9-deficient CD11c^+^ DCs to prime naive CD4^+^ and CD8^+^ T cells was not excluded in this experiment, our data may help evaluate the comparable possibility that TLR2 and/or TLR9-deficient Tip-DCs maintain functional effector CD4^+^ and CD8^+^ T-cell responses in the VT and its draining LNs. In support of this, Ly-6C^hi^ monocytes sorted from the VT of TLR2 and/or TLR9 KO mice after HSV-1 infection were observed to exhibit impaired Ag-presentation to OT-II CD4^+^ T cells compared with Ly-6C^hi^ monocytes from WT mice. However, since Tip-DCs were also observed to exhibit T-cell stimulatory capacity in different infection models (46), the intrinsic ability of Tip-DCs derived from TLR2 and/or TLR9 KO mice to stimulate CD4^+^ and CD8^+^ T cells remains to be investigated.

Another intriguing issue raised in this study was that TLR2/9 directly activate NK cells without the aid of DCs. HSV-1 infection could be recognized by TLR2 and TLR9 expressed in DCs, and thereby induce the production of cytokines, such as IFN-I, IL-12, and IL-15, that play an important role in indirect activation of NK cells (49–51). However, our data support that NK cell activation depends on triggering the TLR2 pathway on NK cells rather than TLR2-depedence in DCs. Indeed, our data are strengthened by a few reports showing that TLR2 signaling on NK cells is involved in their direct activation independent of TLR2 in DCs (33, 41, 54). Of note, UV-inactivated HSV-1 was recently demonstrated to stimulate the expression of CD69, degranulation, migration, and cytokine production in NK cells, partly *via* TLR2/PKC/NF-κB signaling (55), suggesting that certain surface components of UV-inactivated HSV-1 directly activate NK cells. It is conceivable that gH/L and gB trigger the TLR2 pathway for direct NK cell activation (7, 8). However, the role of TLR9 in directly activating NK cells remains obscure because CpG DNA, a TLR9 ligand, failed to directly activate isolated NK cells (56, 57). Most of the NK cell activation by TLR9 ligands is dependent on DCs and/or cytokines produced from DCs. Nevertheless, our results are consistent with very recent results showing that baculovirus directly activates NK cells *via* TLR9, coinciding with the expression of CD69 and promotion of IFN-γ production and cytotoxicity (58). Also, because NK cells were shown to be directly activated with CpG (59), NK cells are likely to be exposed on CpG derived from HSV-1 through intracellular TLR9 at certain stage. HSV-1 is detected in sequence by TLR2/9 in DCs: first, by surface TLR2 interacting with the virions and second, by intracellular TLR9 recognizing the viral genome DNA (10). This sequential recognition of HSV-1 must occur within the same DCs upon direct recognition of the virus and not through activation of bystander DCs (10). Similarly, it is thought that this sequential recognition of HSV-1 by TLR2 and TLR9 occurs in NK cells because TLR2 is expressed on the surface of NK cells, whereas TLR9 has an intracellular localization overlapping with the Golgi apparatus (60, 61). Also, since TLR2 and TLR9 use the same adaptor molecule, MyD88, to transmit signals to a cascade of MAPKs that induces transcriptional expression of inflammatory cytokines (52), dual and sequential recognition of HSV-1 by TLR2 and TLR9 is assumed to amplify their intracellular signal in NK cells. Our data showing that TLR2/9-deficient NK cells drastically and specifically reduced the phosphorylation of p38 MAPK with HSV-1 infection support this speculation. It is likely that there are subtle differences in the activation of MAPKs following direct TLR2 triggering by HSV-1 in NK cells (41, 55). By contrast with the activation of the p38 MAPK pathway in this study, NK cells are directly activated by TLR2 triggering *via* the PI3K/ERK pathway and PKC/NF-κB in vaccinia virus and UV-inactivated HSV-1 infections, respectively (41, 55). This subtle difference in the cascade of intracellular signals for direct NK cell activation by HSV-1 is thought to be derived from the virus strain used in the study, because the utilization of TLR2 and TLR9 depends on HSV strains and specific cell types (10). Ultimately, this direct activation of NK cells by dual TLR2/9 recognition of HSV-1 infection could help to stimulate subsequent CD4^+^ and CD8^+^ T-cell responses in the VT through interacting with CD4^+^ T cells (54) or cross-presentation of apoptotic cell-derived Ag by DCs (62), along with the cooperative contribution of Tip-DCs.

A previous study demonstrated that MyD88, a common adaptor molecule for both TLR2 and TLR9, is fundamental in the immune defense against HSV, as observed by the 100% mortality in MyD88-ablated mice (63). In the context of TLR2 or TLR9 deficiency alone, inoculation with HSV, especially in TLR2 KO mice, led to localized viral replication or starkly contrasting results in mortality, depending on the virus strain and inoculation route (12–16). Our result showed a minor role of TLR2 in conferring protective immunity against mucosal infection with HSV-1. However, dual ablation of TLR2/9 appeared to diminish the protective effects conferred by TLR2 ablation, which may reflect a cooperative role of TLR2 and TLR9. Thus, it is thought that although TLR9 seems to play a more important role than TLR2, both receptors are not only pivotal immune receptors in HSV-1 recognition and control but may also cooperate to generate effective innate and subsequent adaptive immunity in the VT against mucosal infection with HSV-1. Moreover, the diminished survival rate in TLR2/9 DKO mice was closely associated with the reduction of early Ly-6C^hi^ monocyte and NK cell infiltration in the VT. This suggests that dual TLR2/9 recognition of HSV-1 infection contributes to orchestrated mobilization of innate and adaptive immunity-related cells in the VT for the early control of viral replication, thereby preventing CNS-invasion. Although the redundancy of chemokine recognition by immune cells sometimes makes the interpretation of leukocyte infiltration in inflamed tissues difficult, impaired infiltration of Ly-6C^hi^ monocytes and NK cells in the VT of TLR2/9 DKO mice can be explained by the low expression of the CCR2 ligands, CCL2 and CCL7, that play a role in the recruitment of Ly-6C^hi^ monocytes and NK cells (45). Here, one interesting result was that dual ablation of TLR2/9 resulted in massive infiltration of Ly-6G^hi^ granulocytes in the VT, which suggests that TLR2/9 signals may be a negative regulator in the recruitment of Ly-6G^hi^ granulocytes in the VT. Although the role of Ly-6G^hi^ granulocytes in viral infection remains controversial, these cells are believed to be involved in immunopathology (64, 65). IFN-I was recently reported to abrogate the recruitment of Ly-6G^hi^ granulocytes to the ganglia by directly suppressing CXCL2 expression by Ly-6C^hi^ monocytes (66). Conceivably, recognition of HSV-1 infection by specialized cells such as pDCs through TLR9 is expected to produce IFN-I (9). Also, Ly-6C^hi^ monocytes appear to be an important cell population for producing IFN-I *via* TLR2 recognition of HSV-1 infection (34). Therefore, the reduced production of vaginal IFN-I in TLR2 and/or TLR9 KO mice is likely to result in enhanced recruitment of Ly-6G^hi^ granulocytes in the VT. Supporting this speculation, our data revealed that TLR2/9 DKO mice showed greatly increased expression of CXCL2 in the VT. In addition, Ly-6C^hi^ monocytes sorted from the VT of TLR2/9 DKO mice displayed greater expression of IL-23, CXCL1, and CXCL2, which contribute to the recruitment of Ly-6G^hi^ granulocytes in the VT.

Besides TLR2 and TLR9, HSV-1 infection can be recognized by other PRRs such as DNA-dependent activator of IFN-regulatory factors (DAI), IFN-γ-inducible protein 16 (IFI16), and TLR3 (67). Although our data discount the indirect antiviral action generated by these PRRs in activating innate immune cells including monocytes and NK cells, we surmise a coordinated role of TLR2 and TLR9 in directly regulating the differentiation and maturation of infiltrated Ly-6C^hi^ monocytes and NK cells in the VT, thereby contributing to effective and early viral clearance in primary inflamed tissues. Also, although our data provide no evidence for direct correlation between the impaired activation of Tip-DCs and NK cells and the enhanced susceptibility in TLR2/9 DKO, our findings elucidate a detailed TLR2/9-dependent pathway that establishes effective innate and adaptive immune responses in relevant mucosal tissues after natural mucosal infection with HSV-1. To the best of our knowledge, this is the first report that elucidates the detailed role of TLR2/9 in conferring antiviral innate and adaptive immunity in the relevant VT after mucosal infection with HSV-1.

## Ethics Statement

All animal experiments described in the present study were conducted at Chonbuk National University according to the guidelines set by the Institutional Animal Care and Use Committee (IACUC) of Chonbuk National University, and were pre-approved by the Ethical Committee for Animal Experiments of Chonbuk National University (Permission code 2013-0028). The animal research protocol used in this study followed the guidelines set up by the nationally recognized Korea Association for Laboratory Animal Sciences (KALAS). All experimental protocols requiring biosafety were approved by the Institutional Biosafety Committee (IBC) of Chonbuk National University.

## Author Contributions

EU, JC, AP, and SE conceived and designed the research; EU, JC, AP, FH, and SP performed the animal study design, analysis, and interpretation; EU and JC performed *in vitro* experiments; BK and KK provided critical discussion for histopathological examinations and important resources; EU, JC, and SE performed data interpretation and wrote the manuscript. All authors reviewed the manuscript.

## Conflict of Interest Statement

The authors declare that the research was conducted in the absence of any commercial or financial relationships that could be construed as a potential conflict of interest.

## Abbreviations

CNS, central nervous system; DC, dendritic cell; DKO, double knock-out; HSV, herpes simplex virus; KO, knock-out; BM, bone marrow; BMDC, BM-derived DC; dpi, days post-infection; i.vag., intravaginal; OVA, chicken ovalbumin; Tip-DC, TNF-α and iNOS-producing dendritic cell; IFN-I, type I IFN; WT, wild-type.
