# Supplementary material for: Dual TLR2/9 Recognition of Herpes Simplex Virus Infection Is Required for Recruitment and Activation of Monocytes and NK Cells and Restriction of Viral Dissemination to the Central Nervous System
Source: Front Immunol. 2018 Apr 30;9:905. doi: 10.3389/fimmu.2018.00905 (PMC5936768; doi:10.3389/fimmu.2018.00905)
Supplement: Supplementary file 1 [file Presentation_1.PDF]

[Supplementary materials]

Dual TLR2/9 recognition of herpes simplex virus infection is required for recruitment and activation of monocytes and NK cells and restriction of viral dissemination to the CNS

Erdenebileg Uyangaa[1◑], Jin Young Choi[1◑], Ajit Mahadev Patil[1], Ferdaus Mohd Altaf Hossain[1], Seong OK Park[1], Koanhoi Kim[2], Seong Kug Eo[1*]

[1]College of Veterinary Medicine and Bio-Safety Research Institute, Chonbuk National University, Iksan 54596, Republic of Korea
[2]Department of Pharmacology, School of Medicine, Pusan National University, Yangsan 50612, Republic of Korea

Table S1. Antibodies used in flow cytometry and other experiments of this study

Molecule	Clone	Fluorescence dye
CD3ε	145-2C11	FITC
CD4	RM4-5	FITC
CD8α	53-6.7	PE
CD11c	N418	FITC
CD11b	M1/70	PE
CD40	HM40-3	FITC
CD44	IM7	FITC
CD62L	MEL-14	FITC
CD69	H1.2F3	FITC
KLRG1	2F1	FITC
CD80	16-10A1	FITC
CD86	GL1	FITC
CCR2	475301	PE
CXCR2	242216	PE
MHC I (H-2b)	28-14-8	FITC
MHC II (I-Ab)	M5/114.15.2	FITC
CD154(CD40L)	MR1	PE
F4/80	BM8	PE
IFN-γ	XMG1.2	PE
Granzyme-B	NGZB	PE
Gr-1	RB6-8C5	APC
Ly-6G	1A8	APC
Ly-6C	HK1.4	PerCP-Cy5.5
NK1.1	PK136	PE-Cyanine7
CD49b	DX5	APC
IFN- γ	XMG1.2	PerCP-Cy5.5
TNF-α	MP6-XT22	APC
NOS2	CXNFT	APC

Table S2. Specific primers for HSV gB, cytokines, chemokines, and TLRs used in real-time qRT-PCR.

Gene name[a]	Primer sequence (5′-3′)[b]	Position cDNA	Gene Bank ID
**HSV-1gB**	FP: CGT TTC GCA GGT GTG GTT C<br>RP: ATG TCG GTC TCG TGG TCG	507-525<br>678-695	KY_274368.1
**TNF-α**	FP: CGT CGT AGC AAA CCA CCA AG<br>RP: TTG AAG AGA ACC TGG GAG TAG ACA	438-457<br>564-587	NM_013693
**IL-6**	FP: TGG GAA ATC GTG GAA ATG AG<br>FP: CTC TGA AGG ACT CTG GCT TTG	209-228<br>442-462	NM_031168
**iNOS**	FP: AAC GGA GAA CGT GGA TTT G<br>RP: CAG CAC AAG GGG TTT TC	212-231<br>342-358	NM_010927.4
**IFN-α**	FP: TGT CTG ATG CAG CAG GTG G<br>RP: AAG ACA GGG CTC TCC AGA C	367-385<br>514-532	NM_008334.3
**IFN-β**	FP: TCC AAG AAA GGA CGA ACA TTC G<br>RP: TGA GGA CAT CTC CCA CGT CAA	106-121<br>399-419	NM_010510
**IL-23**	FP: CCA GCA GCT CTC TCG GAA TC<br>RP: TCA TAT GTC CCG CTG GTG C	211-230<br>257-275	NM_031252.2
**CCL2**	FP: AAA AAC CTG GAT CGG AAC CAA<br>RP: CGG GTC AAC TTC ACA TTC AAA G	347-367<br>426-447	NM_011333
**CCL3**	FP: CCA AGT CTT CTC AGC GCC AT<br>RP: GAA TCT TCC GGC TGT AGG AGA AG	158-177<br>206-228	NM_011337.2
**CXCL1**	FP: CGC TGC TGC TGG CCA CC<br>RP: GGC TAT GAC TTG GGT TTG GG	101-120<br>245-264	NM_008176.3
**CXCL2**	FP: ATC CAG AGC TTG AGT GTG ACG C<br>RP: AAG GCA AAC TTT TTG ACC GC	194-215<br>264-283	NM_009140.2
**TLR1**	FP: CAA TGT GGA AAC AAC GTG GA<br>RP: TGT AAC TTT GGG GGA AGC TG	933-952<br>1113-1132	NM_030682.2
**TLR2**	FP: AAG AGG AAG CCC AAG AAA GC<br>RP: CGA TGG AAT CGA TGA TGT TG	2234-2253<br>2413-2432	NM_011905.3
**TLR3**	FP: CAC AGG CTG AGC AGT TTG AA<br>RP: TTT CGG CTT CTT TTG ATG CT	2610-2629<br>2780-2799	NM_126166.4
**TLR4**	FP: ACC TGG CTG GTT TAC ACG TC<br>RP: CTG CCA GAG ACA TTG CAG AA	1024-1043<br>1205-1224	NM_021297.3
**TLR5**	FP: AAG TTC CGG GGA ATC TGT TT<br>RP: GCA TAG CCT GAG CCT GTT TC	2316-2335<br>2497-2516	NM_016928.3
**TLR6**	FP: TTC CCA ATA CCA CCG TTC TC<br>RP: CTA TGT GCT GGA GGG TCA CA	743-762<br>924-943	NM_011604.3
**TLR7**	FP: GGA AAT TGC CCT CGA TGT TA<br>RP: CAA AAA TTT GGC CTC CTC AA	1003-1022<br>1220-1239	NM_133211.4
**TLR8**	FP: GGC ACA ACT CCC TTG TGA TTC AT<br>RP: TTG GGT GCT GTT GTT TG	436-455<br>611-627	NM_133212.3
**TLR9**	FP: ACT GAG CAC CCC TGC TTC TA<br>RP: AGA TTA GTC AGC GGC AGG AA	1481-1499<br>1658-1677	NM_031178.2
**β-actin**	FP: TGG AAT CCC TGT GGG ACC ATG AAA C<br>RP: TAA AAC GCA GCT CAG TAA CAG TCC G	885-909<br>1209-1233	NM_007393.3

[a] IL, interleukin; TNF-α, tumor necrosis factor-α; IFN, interferon, TLR, Toll like receptor

[b] FP, forward primer; RP, reverse primer

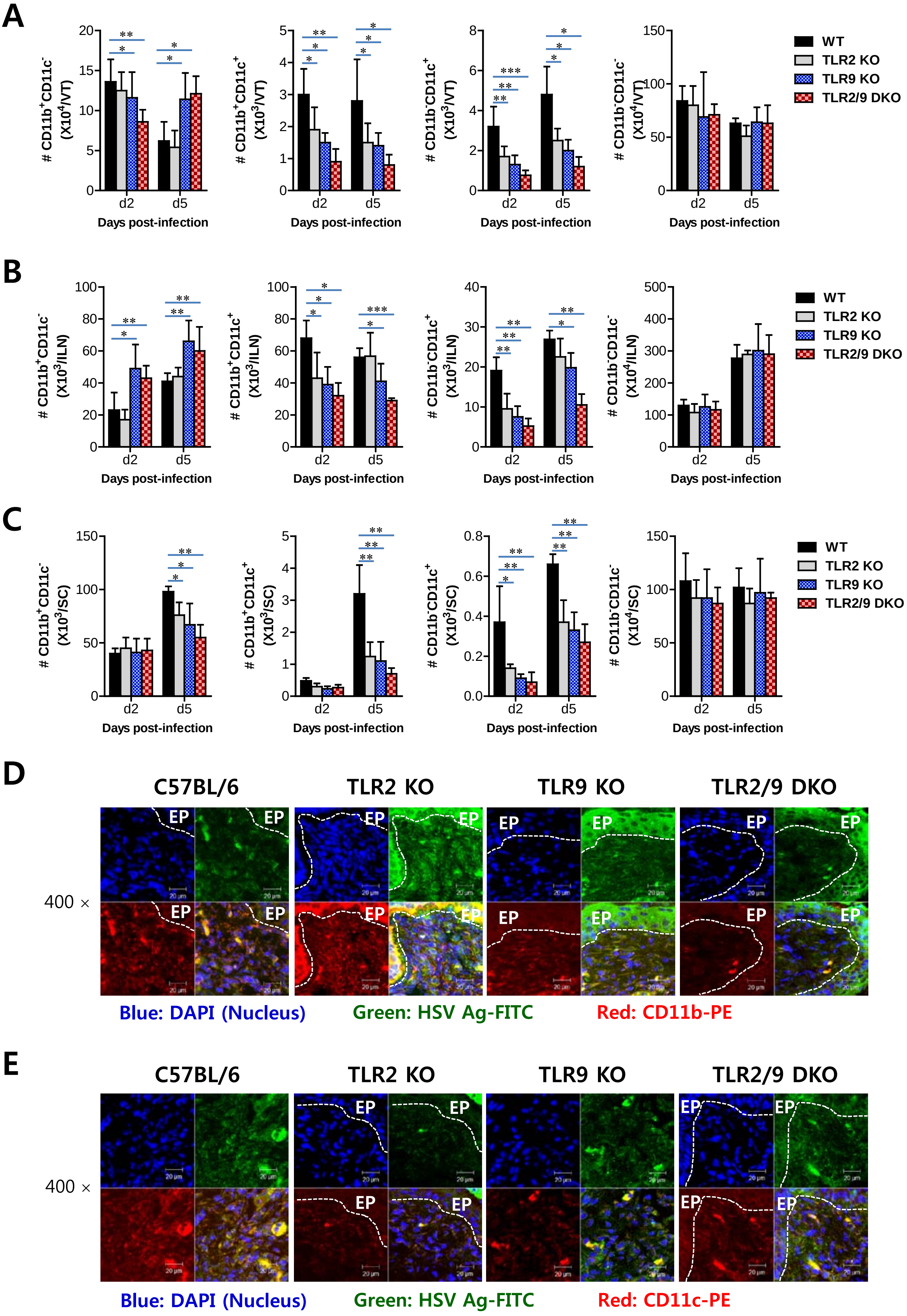

A
B
C
CD11b+CD11c- (X10^4/VT)
CD11b+CD11c+ (X10^3/VT)
CD11b-CD11c+ (X10^3/VT)
CD11b-CD11c- (X10^4/VT)
CD11b+CD11c- (X10^3/ILN)
CD11b+CD11c+ (X10^3/ILN)
CD11b-CD11c+ (X10^3/ILN)
CD11b-CD11c- (X10^4/ILN)
CD11b+CD11c- (X10^3/SC)
CD11b+CD11c+ (X10^3/SC)
CD11b-CD11c+ (X10^3/SC)
CD11b-CD11c- (X10^4/SC)
d2
d5
Days post-infection
WT
TLR2 KO
TLR9 KO
TLR2/9 DKO
D
C57BL/6
TLR2 KO
TLR9 KO
TLR2/9 DKO
400 ×
EP
Blue: DAPI (Nucleus)
Green: HSV Ag-FITC
Red: CD11b-PE
E
C57BL/6
TLR2 KO
TLR9 KO
TLR2/9 DKO
400 ×
EP
Blue: DAPI (Nucleus)
Green: HSV Ag-FITC
Red: CD11c-PE

Figure S1. Infiltration of $CD11^+$ myeloid-derived cells and $CD11c^+$ DCs in inflammatory tissues following mucosal HSV-1 infection. (A-C) The subpopulations of $CD11b^+$ and $CD11c^+$ cells in the vaginal tract, iliac LNs, and spinal cord. Cells were prepared from the vaginal tract (VT, A), iliac LN (ILN, B), and spinal cord (SC, C) with collagenase digestion at 2 and 5 days after mucosal HSV-1 infection (1×10^7 pfu/mouse) and employed to determine the subcellular proportion of $CD11b^+$ and $CD11c^+$ cells using flow cytometric analysis. (D and E) Confocal microscopy for $CD11b^+$ myeloid-derived cells and $CD11c^+$ DCs in the vaginal tract. Sections of the vaginal tract obtained from HSV-infected WT, TLR2 KO, TLR9 KO, and TLR2/9 DKO mice were co-stained for HSV Ag (gB) (green), the nuclear stain DAPI (blue), and the myeloid marker CD11b or DC marker CD11c (red) 2 dpi. Images are representative of sections (400×) from at least four mice. White dot-line shows epithelial layer (EP layer). Data in the bar graphs denote the average ± SEM of the levels derived from at least three independent experiments (n=3-4). Two-way ANOVA followed by Bonferroni *post hoc* testing was conducted in *A*, *B*, and *C*. *, $p< 0.05$; **, $p< 0.01$; ***, $p< 0.001$ comparing levels between the indicated groups.

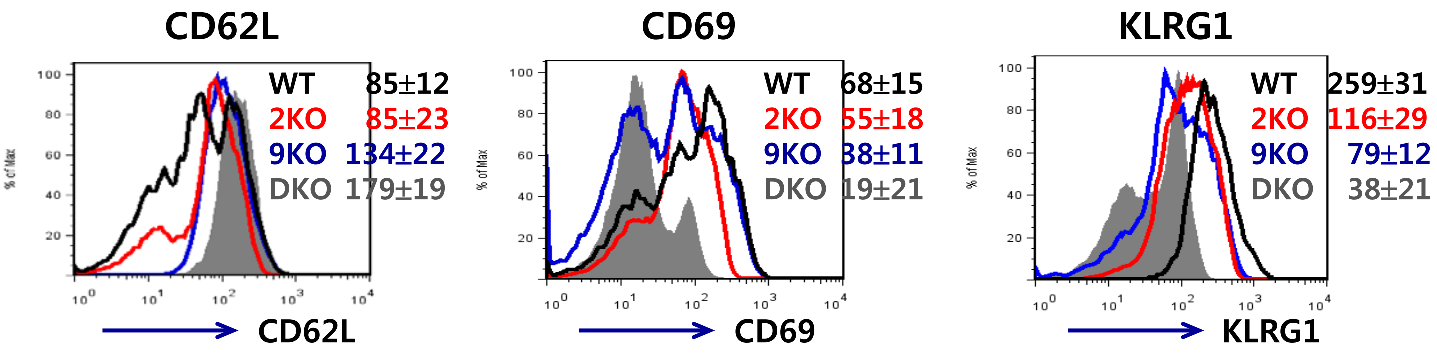

Figure S2. Dual TLR2/9 recognition is required to activate NK cells in the vaginal tract. Cells prepared from the vaginal tract (VT) were subjected to surface staining for activation markers (CD69, CD62L, and KLRG1) of NK cells 2 dpi. The histogram represents the expression of each activation marker after gating on $CD3^-NK1.1^+DX5^+$ NK cells. The values in the histogram represent the average percentage plus SEM of MFI of the indicated activation marker in $CD3^-NK1.1^+DX5^+$ NK cells derived from at least three independent experiments (*n*=4-5).

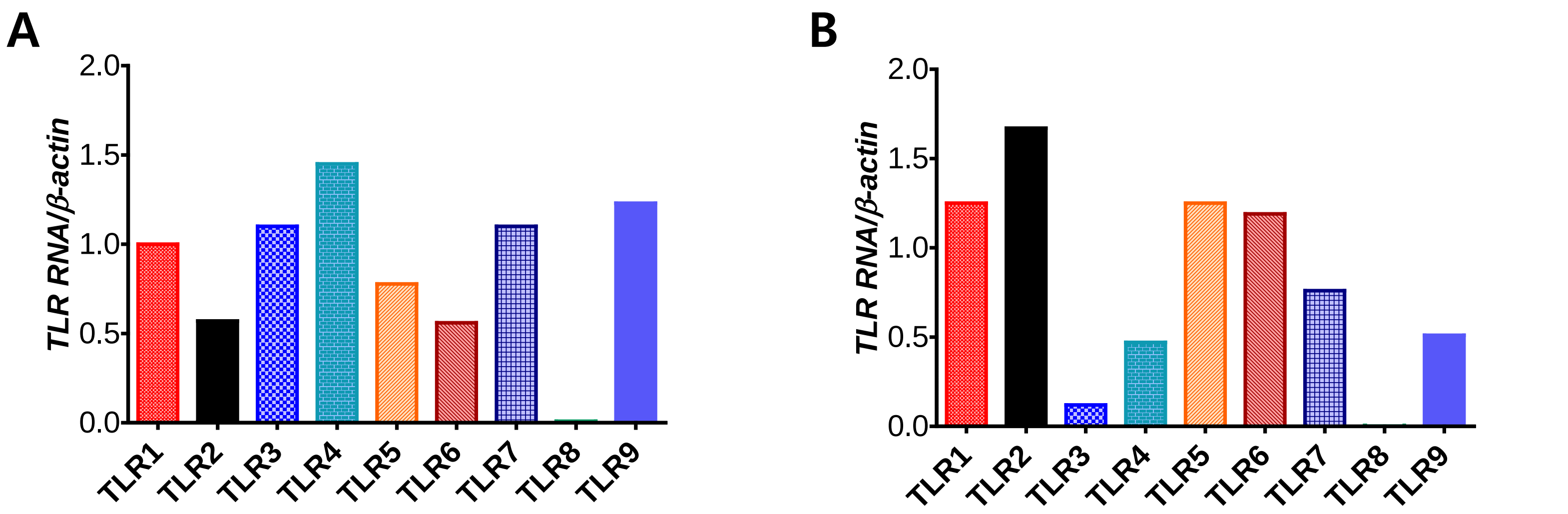

Figure S3. The expression patterns of TLRs in Ly-6C^{hi} and NK cells. (A) Relative expression levels of TLRs in Ly-6C^{hi} monocytes. (B) Relative expression levels of TLRs in NK cells. Total RNA was extracted from Ly-6C^{hi} monocytes and CD3$^-$NK1.1$^+$DX5$^+$ NK cells purified from the spleen of WT mice, and subjected to real-time qRT-PCR. The expression of each TLR is normalized to the housekeeping gene β-actin and displayed as the average of at least four independent samples.
